# Erlotinib regulates short-term memory, tau/Aβ pathology, and astrogliosis in mouse models of AD

**DOI:** 10.3389/fimmu.2024.1421455

**Published:** 2024-10-07

**Authors:** Hyun-ju Lee, Jeong-Woo Hwang, Jieun Kim, A-Ran Jo, Jin-Hee Park, Yoo Joo Jeong, Ji-Yeong Jang, Su-Jeong Kim, Jeong-Heon Song, Hyang-Sook Hoe

**Affiliations:** ^1^ Department of Neural Development and Disease, Korea Brain Research Institute (KBRI), Daegu, Republic of Korea; ^2^ Artificial Intelligence (AI)-based Neurodevelopmental Diseases Digital Therapeutics Group, Korea Brain Research Institute (KBRI), Daegu, Republic of Korea; ^3^ Department of Brain and Cognitive Sciences, Daegu Gyeongbuk Institute of Science & Technology, Daegu, Republic of Korea

**Keywords:** Alzheimer’s disease, erlotinib, tau, amyloid beta, DYRK1A, astrogliosis

## Abstract

**Introduction:**

Erlotinib is an epidermal growth factor receptor (EGFR) inhibitor that is approved by the FDA to treat non-small cell lung cancer (NSCLC). Several membrane receptors, including EGFR, interact with amyloid β (Aβ), raising the possibility that erlotinib could have therapeutic effects on Alzheimer's disease (AD). However, the effects of erlotinib on Aβ/tau-related pathology and cognitive function in mouse models of AD and its mechanisms of action have not been examined in detail.

**Methods:**

To investigate the effects of erlotinib on cognitive function and AD pathology, 3 to 6-month-old PS19 mice and 3 to 3.5-month-old 5xFAD mice and WT mice were injected with vehicle (5% DMSO + 10% PEG + 20% Tween80 + 65% D.W.) or erlotinib (20 mg/kg, i.p.) daily for 14 or 21 days. Then, behavioral tests, Golgi staining, immunofluorescence staining, western blotting ELISA, and real-time PCR were conducted.

**Results and discussion:**

We found that erlotinib significantly enhanced short-term spatial memory and dendritic spine formation in 6-month-old P301S tau transgenic (PS19) mice. Importantly, erlotinib administration reduced tau phosphorylation at Ser202/Thr205 (AT8) and Thr231 (AT180) and further aggregation of tau into paired helical fragments (PHFs) and neurofibrillary tangles (NFTs) in 3-month-old and/or 6-month-old PS19 mice by suppressing the expression of the tau kinase DYRK1A. Moreover, erlotinib treatment decreased astrogliosis in 6-month-old PS19 mice and reduced proinflammatory responses in primary astrocytes (PACs) from PS19 mice. In 3- to 3.5-month-old 5xFAD mice, erlotinib treatment improved short-term spatial memory and hippocampal dendritic spine number and diminished Aβ plaque deposition and tau hyperphosphorylation. Furthermore, erlotinib-treated 5xFAD mice exhibited significant downregulation of astrocyte activation, and treating PACs from 5xFAD mice with erlotinib markedly reduced *cxcl10* (reactive astrocyte marker) and *gbp2* (A1 astrocyte marker) mRNA levels and proinflammatory cytokine mRNA and protein levels. Taken together, our results suggest that erlotinib regulates tau/Aβ-induced AD pathology, cognitive function, and Aβ/tau-evoked astrogliosis and therefore could be a potent therapeutic drug for ameliorating AD symptoms.

## Introduction

Alzheimer’s disease (AD) is a neurodegenerative disorder with a complex pathobiology. The predominant diagnostic symptom of AD is memory impairment induced by amyloid β (Aβ)-containing extracellular plaques and intracellular neurofibrillary tangles (NFTs) composed of misfolded tau/tau aggregates in the brain (1). In addition, the accumulation of Aβ plaques and NFTs in the brain accelerates the transformation of homeostatic microglia and astrocytes to a disease-associated microglia (DAM)/microglial neurodegenerative phenotype (MGnD) and hypertrophic reactive astrocytes (RAs) (2). In a mouse model of AD, DAM/MGnD- and RA-mediated neuroinflammation exacerbates Aβ/tau pathology, thereby contributing to memory impairment (3). Consequently, modulating Aβ/tau pathology, neuroinflammation, and the ensuing cognitive dysfunction, that is, specifically focusing on multiple targets rather than a single target, is a critical strategy for treating AD.

The first-generation epidermal growth factor receptor (EGFR) inhibitor erlotinib is approved by the FDA for the treatment of non-small cell lung cancer (NSCLC) and pancreatic cancer (4). EGFR plays important roles in metabolism and angiogenesis in cancer, and recent studies have suggested that Aβ interacts with several membrane receptors, including EGFR (5–8). The non-canonical stimulation of these receptors by Aβ leads to severe symptoms of AD pathology in hAPP mice and other mouse models of AD (9). Although efforts to ameliorate AD pathology have largely focused on the mechanisms underlying Aβ plaque-mediated cognitive dysfunction, the impact of the EGFR inhibitor erlotinib on tau-mediated AD pathology and cognitive function in tau-overexpressing AD mice has not been fully investigated.

The present study addresses this gap by investigating the effects of erlotinib on cognitive function and AD pathology. We found that erlotinib treatment enhanced short-term spatial memory by promoting hippocampal dendritic spine formation in 6-month-old P301S tau transgenic (PS19) mice. Interestingly, erlotinib treatment significantly decreased tau phosphorylation at Ser202/Thr205 (AT8) and Thr231 (AT180) as well as the formation of paired helical fragments (PHFs) and neurofibrillary tangles (NFTs) by suppressing the expression of the tau kinases DYRK1A, pGSK3α/β, and pCDK5 in 3-month-old and/or 6-month-old PS19 mice. Moreover, erlotinib treatment decreased astrocytic activation and proinflammatory cytokines levels in 6-month-old PS19 mice and in primary astrocytes (PACs) from PS19 mice. We then investigated the effects of erlotinib on learning and memory in 3-month-old 5xFAD mice and found that erlotinib increased short-term spatial memory and hippocampal dendritic spine number. In 3- to 3.5-month-old 5xFAD mice, erlotinib treatment significantly downregulated Aβ plaque deposition and tau hyperphosphorylation. Moreover, erlotinib administration significantly inhibited astrocyte activation in 3.5-month-old 5xFAD mice, and erlotinib-treated PACs from 5xFAD mice exhibited significant downregulation of *cxcl10* (reactive astrocyte marker) and *gbp2* (A1 astrocyte marker) mRNA levels and proinflammatory cytokine mRNA and protein levels. Collectively, these findings suggest that the EGFR inhibitor erlotinib could be a strategic therapeutic drug for ameliorating Aβ/tau pathology, reactive astrogliosis, and memory-related disorders, including AD.

## Materials and methods

### Ethics statement

All experiments were approved by the institutional biosafety committee (IBC) and performed in accordance with approved animal protocols of the Korea Brain Research Institute (KBRI, approval no. IACUC-19-00049, IACUC-22-00044).

### Erlotinib treatment

Erlotinib (Cayman Chemical, Cat no: 10483, Ann Arbor, MI, USA) was dissolved in dimethyl sulfoxide (DMSO) or vehicle (5% DMSO + 10% PEG + 20% Tween80 + 65% D.W.) for *in vitro* or *in vivo* experiments, respectively. For *in vivo* experiments, erlotinib was intraperitoneally (i.p.) administered at a dose of 20 mg/kg, which is lower than the tumor-suppressing dose (25 mg/kg to 100 mg/kg (10)) and the anti-apoptosis dose (80 mg/kg and 100 mg/kg (11, 12)). The use of a dose of 20 mg/kg minimized the toxicity of this anti-cancer drug.

### 5xFAD mice, PS19 mice and WT mice

3 to 3.5-month-old 5xFAD mice (Stock No. 34848-JAX; B6.Cg-Tg (APPSwFlLon,PSEN1*M146L*L286V)6799Vas/Mmjax; Jackson Laboratory, Bar Harbor, ME) and 3- or 6-month-old PS19 mice (Stock No. 008169; B6;C3-Tg (Prnp-MAPT*P301S)PS19Vle/J; Jackson Laboratory) mice were selected as mouse models of AD (13, 14). In 5xFAD mice, five familial AD mutations under the control of the Thy1 promoter (APP^Swe,Lon,Flo^ and PS1^M146L,L286V^) lead to Aβ overexpression. PS19 mice, a model of tauopathy, express the human P301S mutation (one N-terminal insert and four microtubule binding repeats (1N4R)) under the control of the mouse prion protein promoter. Tail genomic DNA was used for genotyping. Male C57BL6/N mice (3.5 months old, 25–30 g) were purchased from Orient-Bio Company (Gyeonggi-do, South Korea). To minimize the effects of hormones, only male mice were included in the behavioral experiments. The mice were housed at 22 ± 2°C, 50 ± 5% humidity, and a 12-h light/dark cycle in a pathogen-free facility with chow and water *ad libitum* and were randomly allocated to the erlotinib or vehicle (control) treatment group.

### RIPA-based soluble and insoluble fractionation of brain tissue

We prepared RIPA buffer-based soluble and insoluble tau protein as previously described with minor modifications (15). Briefly, 6-month-old PS19 mice were perfused with PBS, and the brains were extracted (n = 6-8 per group). The entorhinal cortex and hippocampus were dissected and homogenized in RIPA lysis buffer (Merck Millipore, Billerica, MA, USA) supplemented with a protease and phosphatase inhibitor cocktail (Thermo Scientific, Waltham, MA, USA). The homogenates were incubated at 4°C for 2 h and centrifuged at 20,000 × g at 4°C for 20 min. The supernatant was collected as the RIPA-soluble fraction and stored at −80°C until analysis. The pellet was washed once with 1 M sucrose in RIPA lysis buffer, resuspended in 2% SDS solution (1 mL per gram of tissue), and incubated at room temperature (RT) for 1 h. The suspension was centrifuged at 20,000 × g for 1 min at RT, and the supernatant was collected as the RIPA-insoluble fraction and stored at −80°C until analysis.

### Western blotting

To measure the effects of erlotinib on tau pathology and tau kinases, we conducted western blotting of entorhinal cortex/hippocampal tissue from mice treated with erlotinib (20 mg/kg, i.p.) or vehicle (5% DMSO + 10% PEG + 20% Tween80 + 65% D.W.). The tissues were lysed in RIPA buffer (Merck Millipore, Billerica, MA, USA), and the protein concentration in the lysate was quantified relative to a standard BSA solution. Next, 20 μg of protein was electrophoretically separated on an 8% SDS gel and transferred to a polyvinylidene difluoride (PVDF) membrane. The membrane was blocked with 5% skim milk or 5% BSA at RT for 1 h before incubation overnight with anti-p-EGFR (1:1000, Cell Signaling, Cat No. 2236), anti-AT8 (1:1000, Invitrogen, Cat No. mn1020), anti-p-PHF^Ser396^ (1:1000, Invitrogen, Cat No. 44-752G), anti-p-PHF^Ser404^ (1:1000, Invitrogen, Cat No. 44-758G), anti-Tau5 (1:1000, Invitrogen, AHB0042), anti-DYRK1A (l:1000, Abcam, Cat No. ab180910), anti-p-GSK3α/β (1:1000, Abcam, Cat No. ab75745), anti-p-CDK5 (1:1000, MybioSource, Cat No. MBS2534755), anti-CDK5 (1:1000, Abcam, ab40773) or anti-GAPDH (l:1000, Proteintech, Cat No. 104941-AP) antibodies at 4°C. Next, the membrane was incubated with HRP-conjugated goat anti-mouse or anti-rabbit IgG (both 1:1000, Enzo Life Sciences, Farmingdale, NY, USA) for 1 h. Detection was realized using ECL Western Blotting Detection Reagent (GE Healthcare, Chicago, IL, USA), and images were acquired and analyzed using Fusion Capt Advance software (Vilber Lourmat).

### Immunofluorescence staining

To investigate the effect of erlotinib on AD pathology, 3-month-old and/or 6-month-old PS19 mice, 3 to 3.5-month-old 5xFAD mice or 3.5-month-old WT mice were intraperitoneally (i.p.) injected with 20 mg/kg erlotinib or vehicle (5% DMSO + 10% PEG + 20% Tween80 + 65% D.W.) daily for 14 or 21 days. At the end of the treatment period, the mice were perfused with PBS and fixed with 4% paraformaldehyde. After storage in 4% paraformaldehyde for 24 h at 4°C, the brain tissue was immersed in 30% sucrose in PBS for 72 h; finally, a cryostat microtome (Leica CM1850, Wetzlar, Germany) was used to obtain 30-μm-thick slices. After blocking in 10% normal goat serum (Vector Laboratories, Burlingame, CA, USA) for 2 h at RT, the slices were immunostained with the following primary antibodies at 4°C overnight: anti-p-EGFR (1:100, Invitrogen, Cat No. 44-788G), anti-AT8 (1:200, Invitrogen, Cat No. mn1020), anti-AT100 (1:200, Invitrogen, Cat No. mn1060), anti-AT180 (1:200, Invitrogen, Cat No. mn1040), anti-NFT (1:200, Abcam, Cat No. ab136407), anti-DYRK1A (l:200, Abcam, Cat No. ab180910), anti-p-GSK3α/β (1:200, Abcam, Cat No. ab75745), anti-p-CDK5 (1:200, LS bio, Cat No. LS-C354604-100), anti-6E10 (1:200, Biolegend, Cat No. 803002), anti-4G8 (1:500, Biolegend, Cat No. 800711), anti-GFAP (1:500, Neuromics, Cat No. RA22101; or Invitrogen, Cat No. 13-0300) or anti-Iba-1 (1:500, Wako, Cat No. 019-19741). After washing with PBST buffer, the slices were incubated for 2 h at RT with Alexa 555- or Alexa 488-conjugated secondary antibodies. Finally, the slices were mounted in Vectashield mounting solution containing DAPI (Vector Labs, Burlingame, CA) on glass slides, and images of the immunostained tissue were obtained by fluorescence microscopy (DMi8, Leica Microsystems, Wetzlar, Germany) and analyzed (ImageJ, US National Institutes of Health, Bethesda, MD, USA).

### Y maze

The Y-maze test was performed to assess the effects of erlotinib on short-term and spatial memory in 3-month-old 5xFAD and 6-month-old PS19 mice as previously described (16). The three arms of the Y maze had dimensions of 35 cm x 7 cm x 15 cm and met at 120° angles. A test consisted of a single mouse freely exploring the Y maze for 5 min. Spontaneous alternations during the period of exploration were recorded by a SMART video camera and manually counted. The alternation percentage was calculated by dividing the number of alternations by the number of alternation triads and multiplying by 100.

### Novel object recognition test

The NORT was performed as previously described to evaluate long-term and recognition memory (16). The NORT apparatus consisted of an open-field box with dimensions of 40 cm x 40 cm x 25 cm. The training and testing phases of the NORT were performed at an interval of 24 h. During training, a single mouse explored the box, which contained two identical objects, for 5 min. During testing, the mouse again explored the box for 5 min, but the box contained one familiar object and one novel object. The object locations in the testing phase were counterbalanced among the trials. Odor cues were eliminated between trials by using 70% ethanol to thoroughly clean the box and objects. Each trial was video recorded, and the video was reviewed to manually count the time of exploratory behavior (defined as the mouse pointing its nose toward an object). The novel object preference (%) was calculated from T_Novel_ (novel object exploration time) and T_Familiar_ (familiar object exploration time) as follows: [Object preference (%) = T_Novel_/(T_Familiar_ + T_Novel_) × 100].

### Passive avoidance test

The PAT was performed to analyze aversive memory as described previously (17) with the following modifications. A single mouse was acclimated for 5 min in the passive avoidance chamber (GEMINI Active and Passive Avoidance System, San Diego Instruments, San Diego, CA, USA), which comprised a lit compartment and a dark compartment with a gate between them, and then was returned to its home cage. The next day (day 2, training phase), the mouse was placed in the lit compartment, and after 10 s, the gate opened. Once the mouse entered the dark compartment, the gate closed, and the mouse received an electrical foot shock (3 mA for 3 s). After the foot shock, the mouse was left in the dark compartment for 10 s to enforce an association between the environment and the aversive stimulus and then returned to its home cage. Finally, the mouse was returned to the lit compartment on the following day (day 3, test phase), and after 10 s, the gate was opened. The latency in the lit compartment before the mouse entered the dark compartment was measured with a maximum (cut-off) time of 180 s, and the mouse was returned to its home cage. The mice that did not enter the dark compartment during the training phase (day 2) were excluded from the analysis.

### Golgi staining

After the behavioral tests, Golgi staining of brain tissues from 3-month-old 5xFAD and 6-month-old PS19 mice was performed using the FD Rapid Golgi Stain kit (FD NeuroTechnologies) as previously described (18, 19). Fresh brains were incubated in solutions A and B for 2 weeks at RT and solution C for 24 h at 4°C before slicing using a vibratome (VT1000S; Leica) at a thickness of 150 μm. The number of dendritic spines with a length greater than 20 μm was counted in bright-field microscopy (63× magnification; Axioplan 2; Zeiss) images of pyramidal neurons in cortical layers V and CA1 using ImageJ (NIH).

### PACs from PS19 mice and 5xFAD mice

To investigate the effects of erlotinib on reactive astrogliosis in an *in vitro* model of AD, mixed glial cultures (MGCs) were prepared from postnatal day 1–2 PS19 and 5xFAD mice as previously described with modifications (20, 21). Briefly, tail genomic DNA was used to confirm the genotypes of the pups. Then, whole brains from the PS19 or 5xFAD pups were removed, minced and filtered through 70-μm nylon mesh, and cultured as MGCs in low-glucose DMEM with 10% FBS, 100 U/mL penicillin, and 100 μg/mL streptomycin in a 5% CO_2_ incubator at 37°C for 3 weeks. At day *in vitro* (DIV) 21, primary astrocyte cells (PACs) were isolated from the MGCs by shaking the MGCs in 75 T flasks at 250 rpm on a rotary shaker for 6 h at RT. After discarding the conditioned culture medium, the cells were dissociated by adding trypsin-EDTA (0.25%) and centrifuged at 2000 rpm for 10 min. The pellet containing PACs was collected and seeded at a density of 7×10^5^ cells/well for experiments. At DIV 26, the PACs were treated with vehicle (1% DMSO) or erlotinib (5 μM) for 24 h and then harvested. Total RNA was extracted from the PACs for further analysis.

### Real-time quantitative PCR

To determine whether erlotinib regulates reactive gliosis in an *in vitro* model of AD, total RNA from erlotinib-treated PACs was reverse transcribed to cDNA using the Superscript cDNA Premix Kit II with oligo (dT) primers (GeNetBio, Chungman, Korea). The synthesized cDNA was then used as the template in real-time qPCR with Fast SYBR Green Master Mix (Thermo Fisher Scientific, Waltham, MA, USA) in a QuantStudio 5 Real-Time PCR System (Applied Biosystems, Thermo Fisher Scientific, Waltham, MA, USA). The primer sequences for real-time qPCR are given in **Table 1**. The cycle threshold (Ct) values were normalized to the value for *gapdh*, and the fold change was calculated relative to the control.

**Table 1:** Primers used in real-time qPCR.

Gene		Sequence
gbp2	*Forward*	*5’- GGG GTC ACT GTC TGA CCA CT-3’*
	*Reverse*	*5’- GGG AAA CCT GGG ATG AGA TT-3’*
s100a10	*Forward*	*5’- CAA CGG ACC ACA CCA AAA TGC -3’*
	*Reverse*	*5’- CTG CCT ACT TCT TTC CCT TCT G-3’*
cxcl10	*Forward*	*5’- GCC GTC ATT TTC TGC CTC A-3’*
	*Reverse*	*5’- GCT TCC CTA TGG CCC TCA TT-3’*
il-1β	Forward	5’-TTG ACG GAC CCC AAA AGA TG-3’
	Reverse	5’-AGG ACA GCC CAG GTC AAA G -3’
il-6	Forward *Reverse*	5’-CCA CGG CCT TCC CTA CTT C-3’ *5’-TTG GGA GTG GTA TCC TCT GTG A-3’*
cox-2	Forward *Reverse*	5’CCA CTT CAA GGG AGT CTG GA-3’ *5’-AGT CAT CTG CTA CGG GAG GA-3’*
tnf-α	Forward *Reverse*	5’- TCC AGG CGG TGC CTA TGT-3’ *5’-GCC CCT GCC ACA AGC A-3’*
gapdh	*Forward*	*5’- TGT GTC CGT CGT GGA TCT GA-3’*
	*Reverse*	*5’-CCT GCTTCA CCA CCT TCT TGA -3’*

### Enzyme linked immunosorbent assay

To determine whether erlotinib regulates protein levels of proinflammatory cytokines in PACs from 5xFAD mice, IL-1β, COX-2, IL-6, and TNF-α protein levels were measured by ELISA. For this experiment, PACs from 5xFAD mice were treated with vehicle (1% DMSO) or erlotinib (5 μM) for 24 h. The supernatant of the cell lysate was collected and stored at -80°C until analysis. The protein levels of proinflammatory cytokines in the supernatant were measured using IL-1β, COX-2, IL-6, and TNF-α ELISA kits (IL-1β ELISA kit: 88-7013-88, Invitrogen, Waltham, MA, USA; COX-2 ELISA kit: DYC4198, R&D Systems, Minneapolis, MN, USA; IL-6 ELISA kit: 88-7064-88, Invitrogen, Waltham, MA, USA; TNF-α ELISA kit: 88-7324-88, Invitrogen, Waltham, MA, USA) according to the manufacturer’s instructions as previously described (22).

### Statistical analysis

GraphPad Prism 7 software (GraphPad Software, San Diego, CA, USA) was used to analyze all data. For comparisons between two groups, Student’s t test or the paired t-test was used. One-way ANOVA was used for multiple comparisons. Repeated-measures two-way ANOVA by Bonferroni’s *post hoc* analysis was used for PATs, and p < 0.05 indicated a significant difference. Means ± SEM are presented (*p < 0.05, **p < 0.01, ***p < 0.001).

## Results

### Erlotinib enhances spatial memory and dendritic spine number in PS19 mice

**Figure 1:**
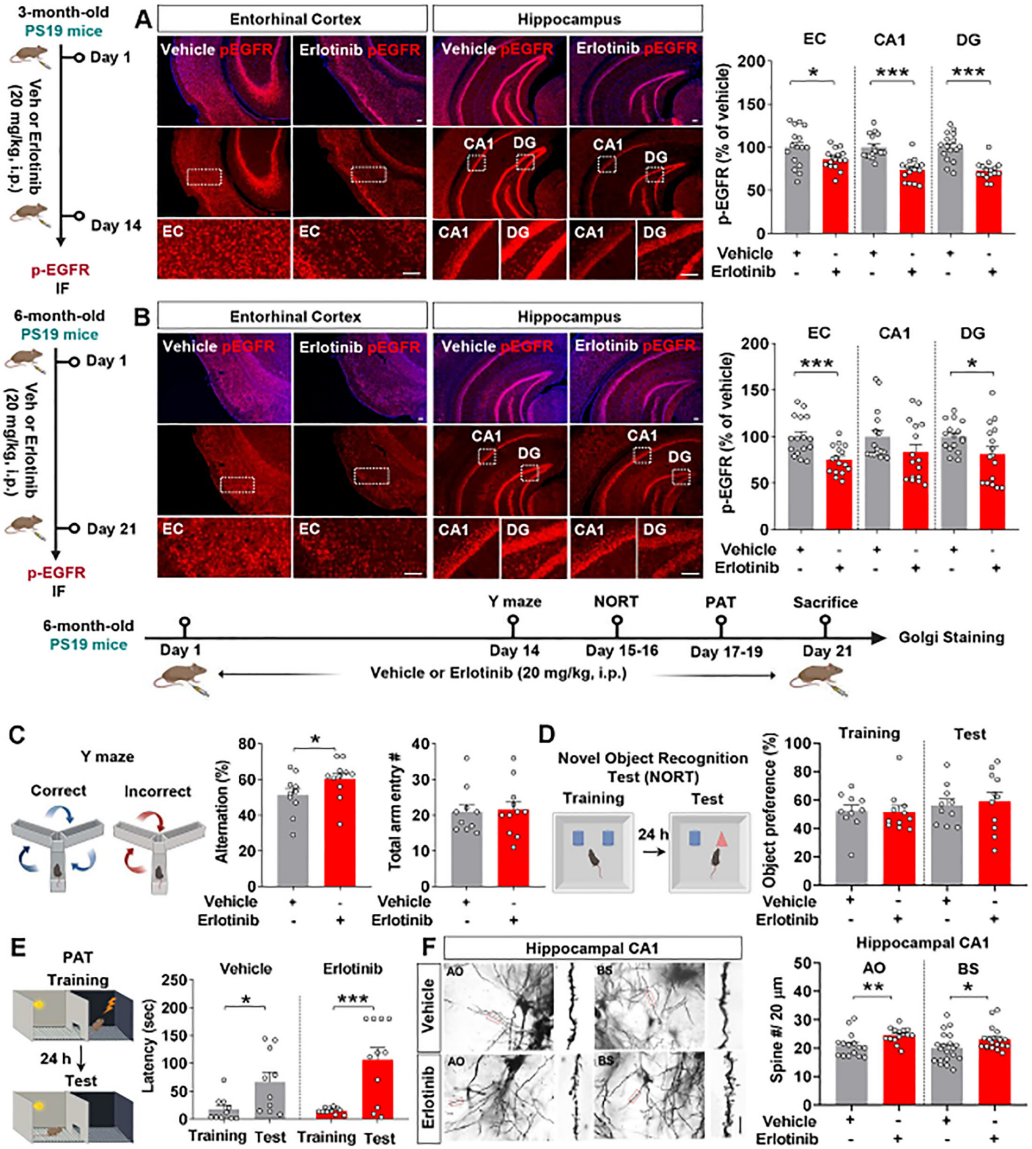
Erlotinib treatment improves spatial memory and dendritic spine number in 6-month-old P301S tau transgenic (PS19) mice. **(A)** 3-month-old PS19 mice were injected with vehicle (5% DMSO + 10% PEG + 20% Tween80 + 65% D.W.) or erlotinib (20 mg/kg, i.p.) daily for 14 days, and immunofluorescence staining was conducted with an anti-p-EGFR antibody (n = 16 brain slices from 4 mice/group). Scale bar = 100 μm. **(B)** 6-month-old PS19 mice were injected with vehicle (5% DMSO + 10% PEG + 20% Tween80 + 65% D.W.) or erlotinib (20 mg/kg, i.p.) daily for 21 days, and immunofluorescence staining was conducted with an anti-p-EGFR antibody (n = 16 brain slices from 4 mice/group). Scale bar = 100 μm. **(C–E)** 6-month-old PS19 mice were injected with vehicle (5% DMSO + 10% PEG + 20% Tween80 + 65% D.W.) or erlotinib (20 mg/kg, i.p.) daily for 21 days, and the Y-maze test, NORT, and PAT were conducted (n = 10-11 mice/group). **(F)** After the behavioral tests, Golgi staining of the hippocampus of 6-month-old PS19 mice was performed (n = 15-20 brain slices from 4 mice/group);. Scale bar = 10 μm. *p < 0.05, **p < 0.01, and ***p < 0.001.

A previous study demonstrated that inhibition of EGFR improves spatial memory in APP/PS1 mice, a mouse model of AD (9). However, the effects of erlotinib on cognitive function have not been fully elucidated in a tau-overexpressing mouse model of AD. Prior to behavioral tests, the effect of erlotinib on EGFR phosphorylation, an on-target of erlotinib, was assessed. For these experiments, 3-month-old PS19 mice were treated with vehicle (5% DMSO + 10% PEG + 20% Tween80 + 65% D.W.) or erlotinib (20 mg/kg, i.p.) daily for 14 days, and immunofluorescence staining was conducted with an anti-p-EGFR antibody. Erlotinib treatment significantly suppressed EGFR phosphorylation in the entorhinal cortex and hippocampus in PS19 mice (**Figure 1A**).

We then examined whether erlotinib alters EGFR phosphorylation in an aged mouse model of AD. Six-month-old PS19 mice were treated with vehicle (5% DMSO + 10% PEG + 20% Tween80 + 65% D.W.) or erlotinib (20 mg/kg, i.p.) daily for 21 days, and immunofluorescence staining was conducted with an anti-p-EGFR antibody. Erlotinib treatment significantly downregulated EGFR phosphorylation in the entorhinal cortex and hippocampal DG region in 6-month-old PS19 mice (**Figure 1B**).

Next, we investigated whether erlotinib regulates learning and memory in 6-month-old PS19 mice. For these experiments, 6-month-old PS19 mice were treated with vehicle (5% DMSO + 10% PEG + 20% Tween80 + 65% D.W.) or erlotinib (20 mg/kg, i.p.) daily for 21 days, and behavior experiments were conducted on days 14–19. Erlotinib treatment significantly increased the alternation % in the Y-maze test compared with vehicle treatment (**Figure 1C**). However, erlotinib administration did not alter recognition memory in the NORT nor associative aversive memory in the PAT compared with vehicle treatment in 6-month-old PS19 mice (**Figures 1D, E**).

Next, to determine whether erlotinib improves short-term spatial memory by regulating dendritic spine formation, 6-month-old PS19 mice were treated with vehicle (5% DMSO + 10% PEG + 20% Tween80 + 65% D.W.) or erlotinib (20 mg/kg, i.p.) daily for 21 consecutive days, and Golgi staining of brain slices was conducted. Importantly, dendritic spine number in the hippocampal apical oblique (AO) and basal shaft (BS) regions was significantly increased in erlotinib-treated PS19 mice compared with vehicle-treated PS19 mice (**Figure 1F**), indicating that erlotinib improves hippocampal-dependent spatial memory by promoting hippocampal dendritic spine formation in 6-month-old PS19 mice. Collectively, these results suggest that administration of the EGFR inhibitor erlotinib improves tau-mediated cognitive impairments in PS19 mice by increasing hippocampal spinogenesis.

### Erlotinib downregulates tau hyperphosphorylation in 3-month-old and 6-month-old PS19 mice

**Figure 2:**
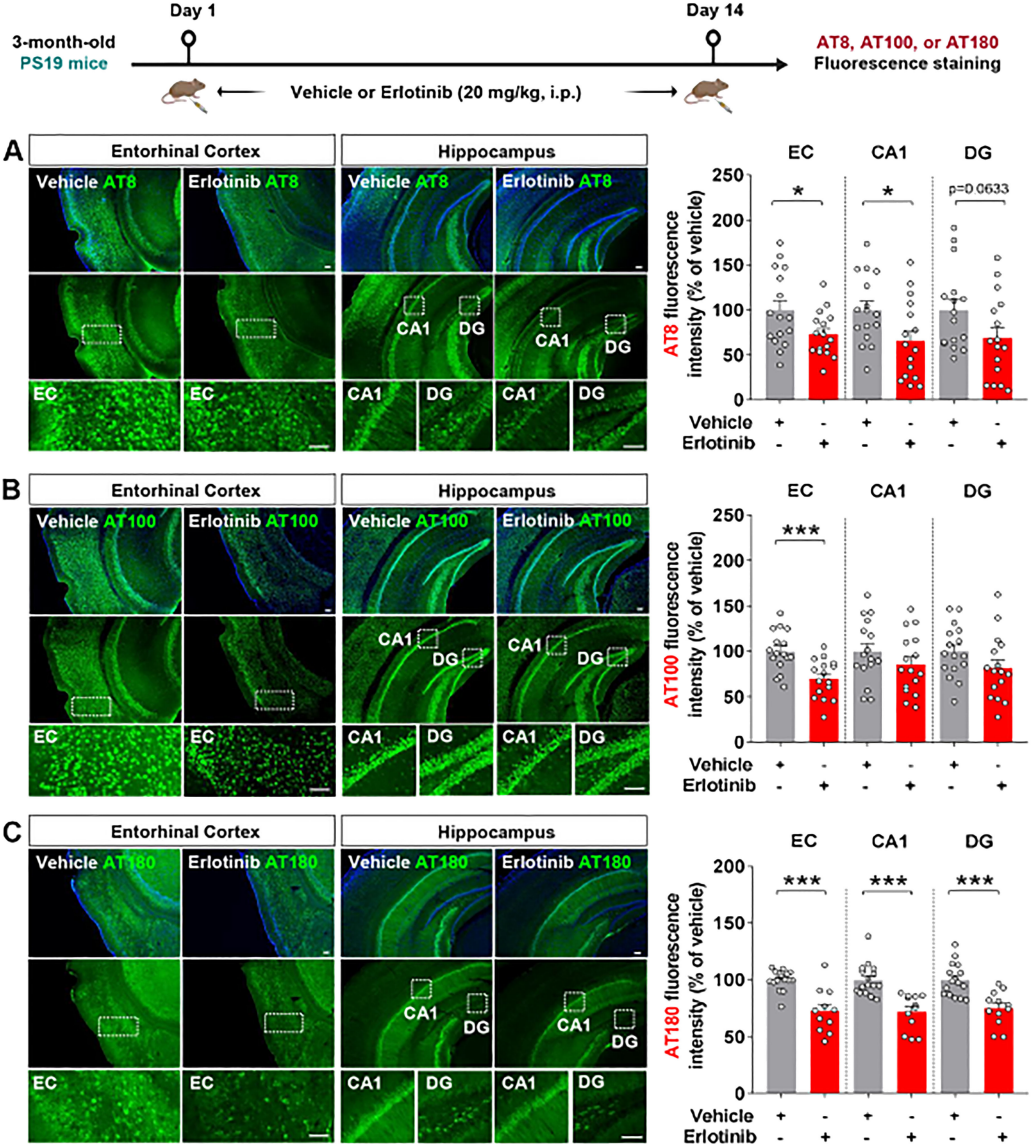
Erlotinib treatment decreases tau hyperphosphorylation in 3-month-old P301S tau transgenic (PS19) mice. **(A–C)** 3-month-old PS19 mice were injected with vehicle (5% DMSO + 10% PEG + 20% Tween80 + 65% D.W.) or erlotinib (20 mg/kg, i.p.) daily for 14 days, and immunofluorescence staining was conducted with anti-AT8 **(A)**, anti-AT100 **(B)**, or anti-AT180 **(C)** antibodies (n = 11-16 brain slices from 3-4 mice/group). Scale bar = 100 μm. *p < 0.05, ***p < 0.001.

Since erlotinib improved cognitive function in PS19 mice, we investigated whether erlotinib modulates tau pathology in younger and aged transgenic PS 19 mice. First, 3-month-old PS19 mice were administered vehicle (5% DMSO + 10% PEG + 20% Tween80 + 65% D.W.) or erlotinib (20 mg/kg, i.p.) daily for 14 days, and immunofluorescence staining of brain slices was conducted with anti-AT8, anti-AT100, and anti-AT180 antibodies. Erlotinib treatment significantly decreased tau phosphorylation at residues Ser202/Thr205 (AT8) in the entorhinal cortex and hippocampal CA1 regions (**Figure 2A**). In addition, erlotinib administration significantly decreased tau phosphorylation at residues Thr212/Ser214 (AT100) in the entorhinal cortex but not in the hippocampus (**Figure 2B**). Furthermore, erlotinib treatment significantly decreased tau phosphorylation at residue Thr231 (AT180) in the entorhinal cortex and hippocampus in 3-month-old PS19 mice (**Figure 2C**).

**Figure 3:**
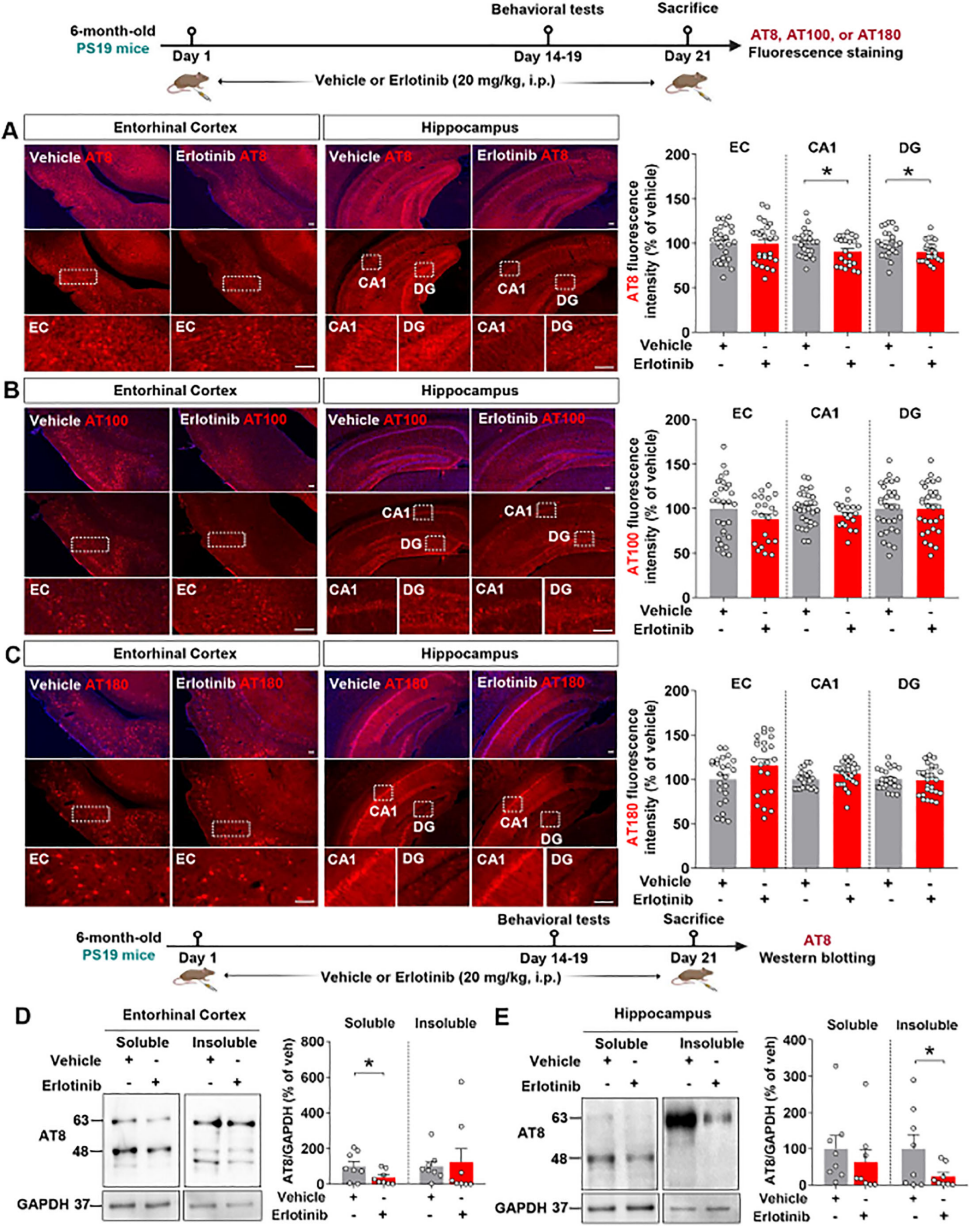
Erlotinib treatment downregulates tau phosphorylation at Ser202/Thr205 (AT8) in 6-month-old P301S tau transgenic (PS19) mice. **(A–C)** 6-month-old PS19 mice were injected with vehicle (5% DMSO + 10% PEG + 20% Tween80 + 65% D.W.) or erlotinib (20 mg/kg, i.p.) daily for 21 days, and brain slices were immunostained with **(A)** anti-AT8, **(B)** anti-AT100, and **(C)** anti-AT180 antibodies (n = 19–30 brain slices from 4-5 mice/group). **(D, E)** 6-month-old PS19 mice were injected with vehicle (5% DMSO + 10% PEG + 20% Tween80 + 65% D.W.) or erlotinib (20 mg/kg, i.p.) daily for 21 days, and immunoblot analysis of the entorhinal cortex **(D)** and hippocampus **(E)** was conducted with an anti-AT8 antibody (n = 8 mice/group). Scale bar = 100 μm. *p < 0.05.

To examine the effects of erlotinib on tau hyperphosphorylation in aged PS19 mice, 6-month-old PS19 mice were administered vehicle (5% DMSO + 10% PEG + 20% Tween80 + 65% D.W.) or erlotinib (20 mg/kg, i.p.) daily for 21 days, and immunofluorescence staining of brain slices was conducted with anti-AT8, anti-AT100, and anti-AT180 antibodies. Importantly, erlotinib treatment significantly decreased tau phosphorylation at Ser202/Thr205 (AT8) in the hippocampus but not in the entorhinal cortex in 6-month-old PS19 mice (**Figure 3A**). In addition, administration of erlotinib did not alter tau phosphorylation at Thr212/Ser214 (AT100) and Thr231 (AT180) in the entorhinal cortex and hippocampus in 6-month-old PS19 mice (**Figures 3B, C**).

To confirm the immunofluorescence staining results, we conducted western blotting of the soluble and insoluble fractions of brain tissues with an anti-AT8 antibody. This analysis showed that erlotinib treatment significantly reduced tau phosphorylation at Ser202/Thr205 (AT8) in the RIPA-soluble fraction but not in the RIPA-insoluble fraction of the entorhinal cortex of 6-month-old PS19 mice (**Figure 3D**). Additionally, erlotinib administration significantly suppressed tau phosphorylation at Ser202/Thr205 (AT8) in the RIPA-insoluble fraction but not in the RIPA-soluble fraction of the hippocampus in 6-month-old PS19 mice (**Figure 3E**). These data indicate that erlotinib selectively regulates tau hyperphosphorylation in PS19 mice.

### Erlotinib reduces PHF and NFT formation in 6-month-old PS19 mice

**Figure 4:**
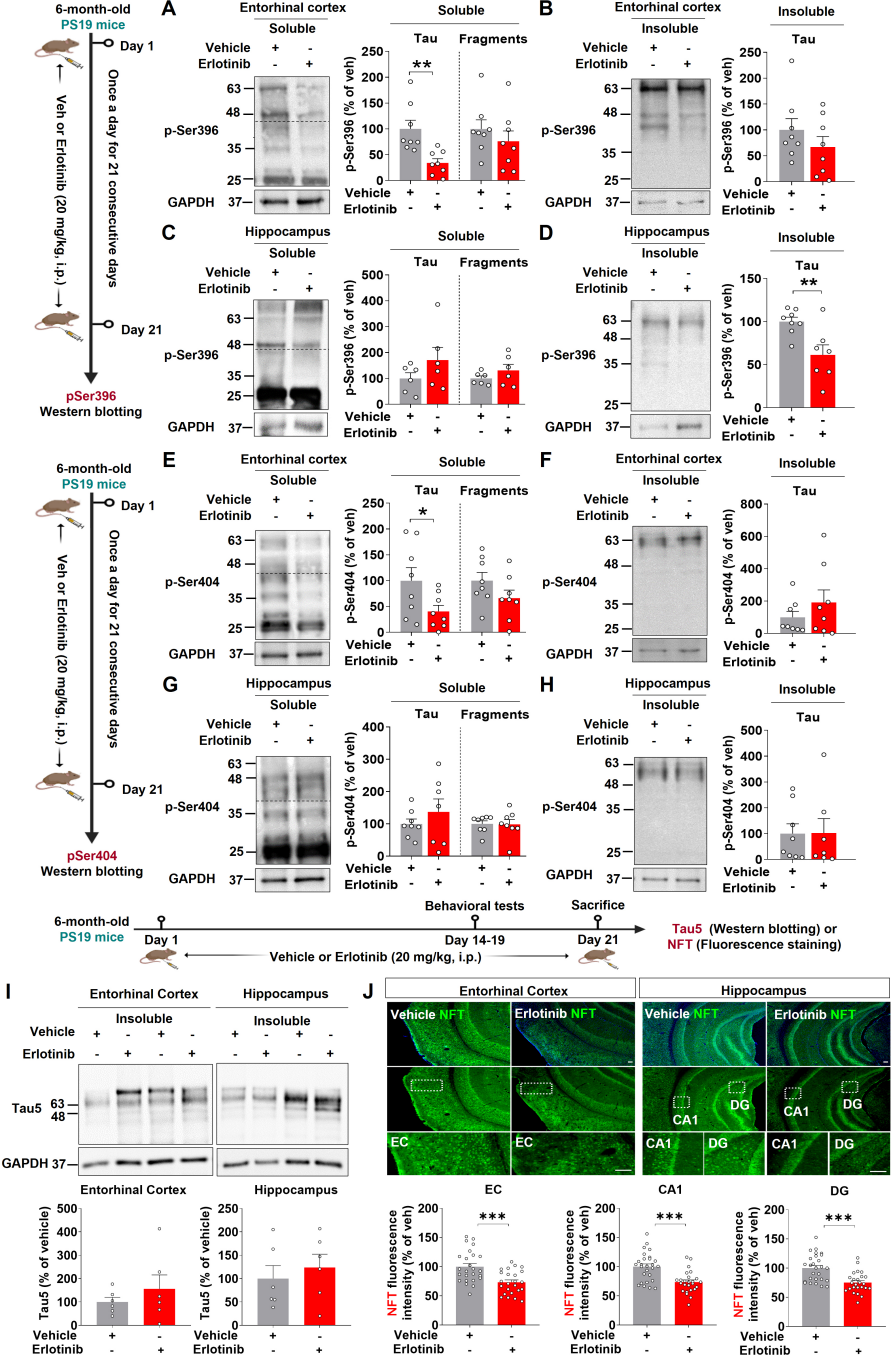
Erlotinib treatment reduces the formation of neurofibrillary tangles (NFTs) and paired helical fragments (PHFs) in 6-month-old P301S tau transgenic (PS19) mice. **(A–D)** 6-month-old PS19 mice were injected with vehicle (5% DMSO + 10% PEG + 20% Tween80 + 65% D.W.) or erlotinib (20 mg/kg, i.p.) daily for 21 days, and immunoblot analysis of the entorhinal cortex **(A, B)** and hippocampus **(C, D)** was conducted with an anti-p-PHF^Ser396^ antibody (n = 6-8 mice/group). **(E–H)** 6-month-old PS19 mice were injected with vehicle (5% DMSO + 10% PEG + 20% Tween80 + 65% D.W.) or erlotinib (20 mg/kg, i.p.) daily for 21 days, and immunoblot analysis of the entorhinal cortex **(E, F)** and hippocampus **(G, H)** was conducted with an anti-p-PHF^Ser404^ antibody (n = 7-8 mice/group). **(I)** 6-month-old PS19 mice were injected with vehicle (5% DMSO + 10% PEG + 20% Tween80 + 65% D.W.) or erlotinib (20 mg/kg, i.p.) daily for 21 days, and immunoblot analysis of the entorhinal cortex and hippocampus was conducted with an anti-Tau5 antibody (n = 6 mice/group). **(J)** 6-month-old PS19 mice were injected with vehicle (5% DMSO + 10% PEG + 20% Tween80 + 65% D.W.) or erlotinib (20 mg/kg, i.p.) daily for 21 days, and immunofluorescence staining was conducted with an anti-NFT antibody (n = 24-27 brain slices from 5 mice/group). Scale bar = 100 μm. *p < 0.05, **p < 0.01, ***p < 0.001.

In PS19 mice, tau filament development begins at 6 months of age (23). We therefore examined the effects of erlotinib treatment on PHF and NFT formation in 6-month-old PS19 mice. Six-month-old PS19 mice were treated with vehicle (5% DMSO + 10% PEG + 20% Tween80 + 65% D.W.) or erlotinib (20 mg/kg, i.p.) daily for 21 days, and RIPA-soluble and -insoluble fractions of brain tissues were obtained. The fractions were then analyzed by immunoblotting with anti-PHF-1 antibodies, including antibodies against p-PHF^Ser396^ and p-PHF^Ser404^. Erlotinib treatment significantly reduced RIPA-soluble p-PHF^Ser396^ levels (45– 70 kDa) but did not alter the levels of RIPA-soluble tau fragments (20–35 kDa) and RIPA-insoluble p-PHF^Ser396^ in the entorhinal cortex in 6-month-old PS19 mice (**Figures 4A, B**). In the hippocampus, erlotinib treatment significantly diminished RIPA-insoluble p-PHF^Ser396^ levels but did not alter the levels of RIPA-soluble p-PHF^Ser396^ and tau fragments (**Figures 4C, D**). Similar to the results for p-PHF^Ser396^, erlotinib treatment significantly decreased RIPA-soluble p-PHF^Ser404^ levels in the entorhinal cortex in 6-month-old PS19 mice but not RIPA-soluble tau fragments and RIPA-insoluble p-PHF^Ser404^ levels (**Figures 4E, F**). In the hippocampus, erlotinib treatment did not alter the levels of RIPA-soluble and RIPA-insoluble p-PHF^Ser404^ levels in 6-month-old PS19 mice (**Figures 4G, H**). Furthermore, we found that erlotinib did not alter total Tau levels (detected by Tau5) in the RIPA-insoluble fractions of the entorhinal cortex and hippocampus in 6-month-old PS19 mice (**Figure 4I**).

We further confirmed these results by performing immunofluorescence staining of brain slices with an anti-NFT antibody that recognizes a phosphorylation-independent epitope in residues 404–441 (human). Erlotinib treatment significantly reduced NFT levels in the entorhinal cortex and hippocampal CA1 and DG regions (**Figure 4J**). Collectively, these data suggest that the EGFR inhibitor erlotinib alleviates tau aggregation in 6-month-old PS19 mice.

### Erlotinib diminishes tau kinase activity/levels in 3-month-old and 6-month-old PS19 mice

**Figure 5:**
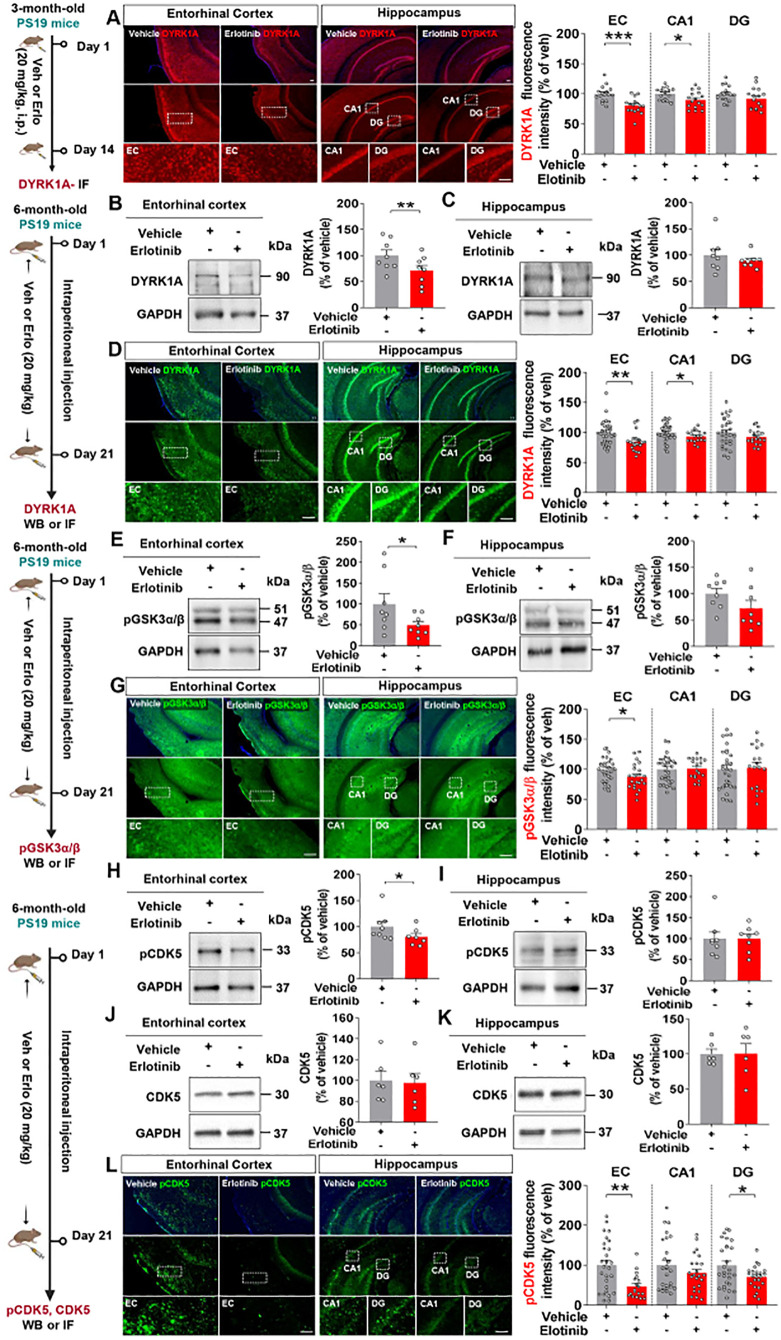
Erlotinib treatment decreases tau kinase levels/activity in 3-month-old and 6-month-old P301S tau transgenic (PS19) mice. **(A)** 3-month-old PS19 mice were injected with vehicle (5% DMSO + 10% PEG + 20% Tween80 + 65% D.W.) or erlotinib (20 mg/kg, i.p.) daily for 14 days, and immunofluorescence staining was conducted with an anti-DYRK1A antibody (n = 16 brain slices from 4 mice/group). **(B, C, E, F, H–K)** 6-month-old PS19 mice were injected with vehicle (5% DMSO + 10% PEG + 20% Tween80 + 65% D.W.) or erlotinib (20 mg/kg, i.p.) daily for 21 days, and brain slices from the entorhinal cortex and hippocampus were immunoblotted with anti-DYRK1A **(B, C)**, anti-pGSK3α/β **(E, F)**, anti-pCDK5 **(H, I)**, and anti-CDK5 antibodies **(J, K)** (n = 6-8 mice/group). **(D, G, L)** 6-month-old PS19 mice were injected with vehicle (5% DMSO + 10% PEG + 20% Tween80 + 65% D.W.) or erlotinib (20 mg/kg, i.p.) daily for 21 days, and immunofluorescence staining was conducted with anti-DYRK1A **(D)**, anti-p-GSK3α/β **(G)**, and anti-p-CDK5 antibodies **(L)** (n = 18-27 brain slices from 4-5 mice/group). Scale bar = 100 μm. *p < 0.05, **p < 0.01, ***p < 0.001.

Since erlotinib treatment suppressed tau pathology in 3-month-old and 6-month-old PS19 mice, we analyzed tau kinase levels to examine the mechanisms by which erlotinib regulates tau hyperphosphorylation. Three-month-old PS19 mice were administered vehicle (5% DMSO + 10% PEG + 20% Tween80 + 65% D.W.) or erlotinib (20 mg/kg, i.p.) daily for 14 days, and immunofluorescence staining of brain slices was conducted with an anti-DYRK1A antibody. Erlotinib administration significantly reduced DYRK1A fluorescence intensity in the entorhinal cortex and hippocampal CA1 region in 3-month-old PS19 mice but had no effect on DYRK1A fluorescence intensity in the hippocampal DG region (**Figure 5A**).

We then investigated the effects of erlotinib on tau kinase levels and activity in aged PS19 mice. Six-month-old PS19 mice were administered vehicle (5% DMSO + 10% PEG + 20% Tween80 + 65% D.W.) or erlotinib (20 mg/kg, i.p.) daily for 21 days, and western blotting of brain lysates was conducted with an anti-DYRK1A antibody. Erlotinib significantly reduced DYRK1A levels in the entorhinal cortex but not in the hippocampus in 6-month-old PS19 mice (**Figures 5B, C**).

To confirm these findings, we performed immunofluorescence staining of brain slices, which showed that erlotinib treatment significantly downregulated DYRK1A immunofluorescence intensity in the entorhinal cortex and hippocampal CA1 region compared with vehicle treatment (**Figure 5D**). In addition, erlotinib administration significantly reduced the level of the tau kinase p-GSK3α/β in the entorhinal cortex but not in the hippocampus in 6-month-old PS19 mice, as assessed by immunoblot analysis and immunofluorescence staining (**Figures 5E–G**). Moreover, erlotinib treatment significantly suppressed the level of the tau kinase p-CDK5 in the entorhinal cortex in 6-month-old PS19 mice as assessed by immunoblot analysis (**Figures 5H, I**). However, erlotinib treatment did not alter total CDK5 levels in 6-month-old PS19 mice (**Figures 5J, K**).

Finally, to further verify our findings, we conducted immunofluorescence staining and found that erlotinib treatment significantly downregulated tau kinase p-CDK5 immunostaining intensity in the entorhinal cortex and hippocampal DG regions in 6-month-old PS19 mice (**Figure 5L**). These data suggest that erlotinib suppresses tau phosphorylation by downregulating tau kinases in 3-month-old and 6-month-old PS19 mice.

### Erlotinib reduces astrogliosis and/or proinflammatory cytokine levels in 6-month-old PS19 mice and PACs from PS19 mice

**Figure 6:**
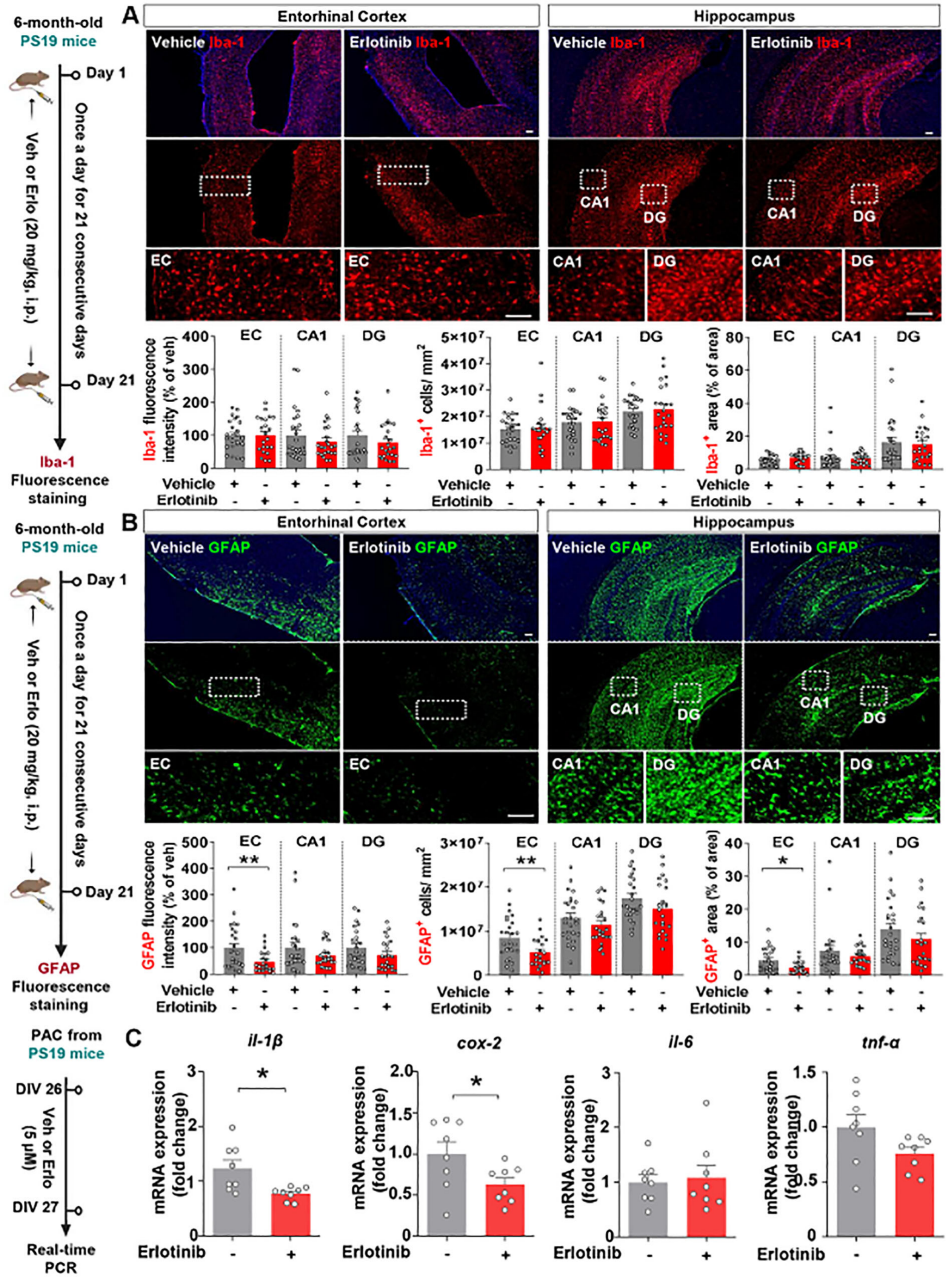
Erlotinib treatment significantly reduces astrocyte activation and proinflammatory cytokine levels in PS19 mice and in primary astrocytes (PACs) from PS19 mice. **(A, B)** 6-month-old P301S tau transgenic (PS19) mice were injected with vehicle (5% DMSO + 10% PEG + 20% Tween80 + 65% D.W.) or erlotinib (20 mg/kg, i.p.) daily for 21 days, and immunofluorescence staining was conducted with anti-Iba-1 **(A)** and anti-GFAP **(B)** antibodies (n = 22 - 26 brain slices from 5 mice/group). **(C)** PACs from PS19 mice were treated with vehicle (1% DMSO) or erlotinib (5 μM) for 24 h, and the relative mRNA levels of the indicated genes were analyzed by real-time PCR (n = 8/group). Scale bar = 100 μm. *p<0.05, **p < 0.01.

Given the effects of erlotinib on tau-induced cognitive impairment and tau aggregation, we next investigated whether erlotinib alters tau-mediated neuroinflammatory responses. Six-month-old PS19 mice were administered vehicle (5% DMSO + 10% PEG + 20% Tween80 + 65% D.W.) or erlotinib (20 mg/kg, i.p.) daily for 21 days, and immunofluorescence staining of brain slices was performed with anti-GFAP (an astrogliosis marker) and anti-Iba-1 (a microgliosis marker) antibodies. Erlotinib treatment did not alter Iba-1 fluorescence intensity, Iba-1-positive cell number, or Iba-1-positive area in the brain in 6-month-old PS19 mice (**Figure 6A**). However, erlotinib treatment significantly reduced GFAP fluorescence intensity, GFAP-positive cell number, and GFAP-positive area in the entorhinal cortex but not the hippocampus in 6-month-old PS19 mice (**Figure 6B**). These data indicate that erlotinib downregulates astrogliosis but has no effects on microgliosis in 6-momth-old PS19 mice.

To further analyze the effect of erlotinib on astrocytic activation *in vitro*, PACs from PS19 mice were treated with vehicle (1% DMSO) or erlotinib (5 μM) for 24 h, and real-time PCR was conducted. Interestingly, we found that erlotinib treatment selectively downregulated *il-1β* and *cox-2* mRNA levels in PACs from PS19 mice compared to vehicle treatment, whereas *il-6* and *tnf-α* mRNA levels were not altered by erlotinib treatment (**Figure 6C**). Collectively, these data suggest that erlotinib suppresses astrogliosis and proinflammatory responses in 6-month-old PS19 mice and/or PACs from PS19 mice.

### Erlotinib modulates spatial memory, hippocampal dendritic spine number and Aβ pathology in 3- to 3.5-month-old 5xFAD mice

**Figure 7:**
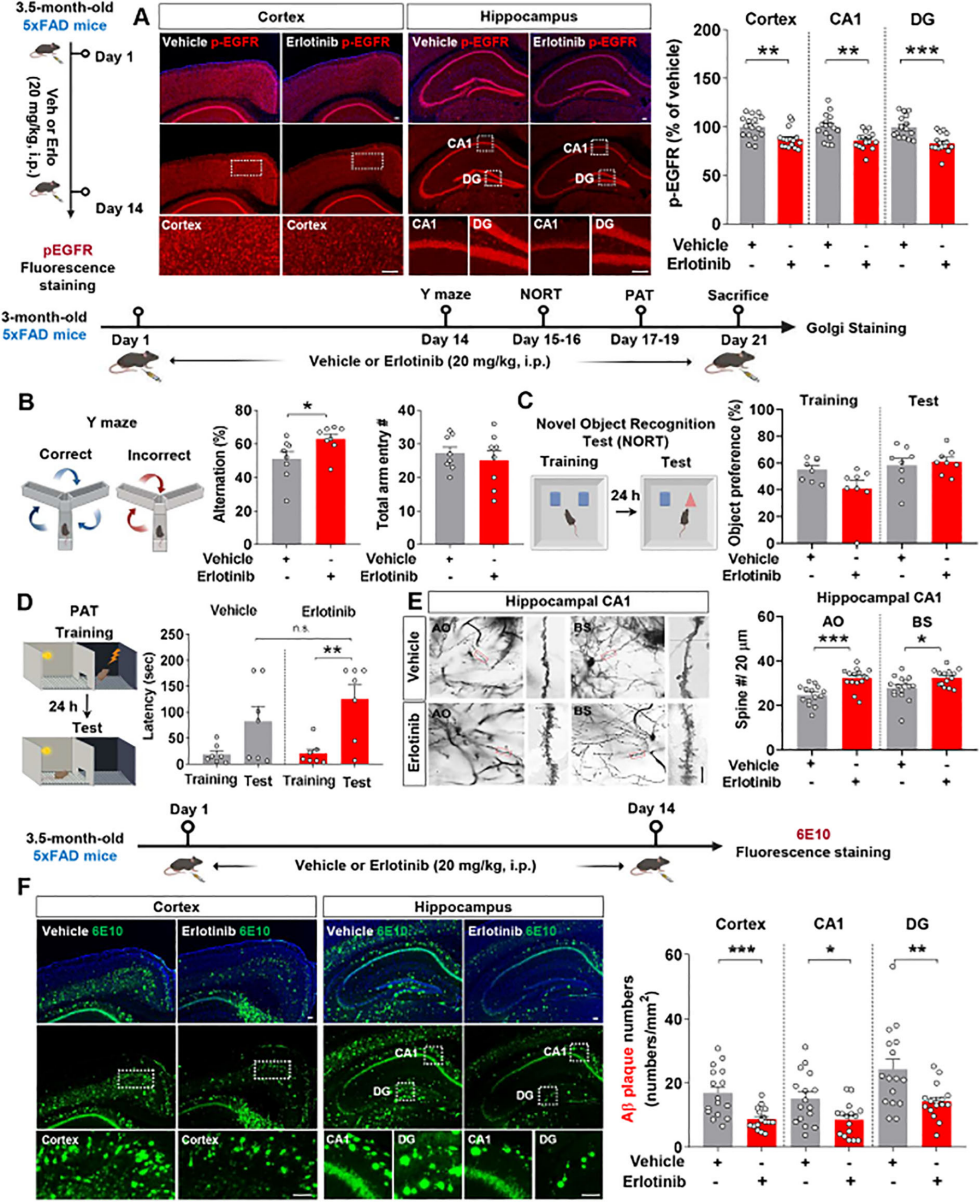
Erlotinib treatment enhances spatial memory and hippocampal dendritic spine number while decreasing Aβ plaque number in 5xFAD mice. **(A)** 3.5-month-old 5xFAD mice were injected with vehicle (5% DMSO + 10% PEG + 20% Tween80 + 65% D.W.) or erlotinib (20 mg/kg, i.p.) daily for 14 days, and immunofluorescence staining was performed with an anti-p-EGFR antibody (n = 16 brain slices from 4 mice/group). **(B–D)** 3-month-old 5xFAD mice were injected with vehicle (5% DMSO + 10% PEG + 20% Tween80 + 65% D.W.) or erlotinib (20 mg/kg, i.p.) daily for 21 days, and the Y-maze test **(B)**, NORT **(C)** and PAT **(D)** were conducted (n = 7-8 mice/group). **(E)** After the behavioral tests, Golgi staining of the hippocampus was performed (n = 14-15 brain slices from 3 mice/group). Scale bar = 10 μm. **(F)** 3.5-month-old 5xFAD mice were injected with vehicle (5% DMSO + 10% PEG + 20% Tween80 + 65% D.W.) or erlotinib (20 mg/kg, i.p.) daily for 14 days, and immunofluorescence staining was performed with an anti-6E10 antibody (n = 16 brain slices from 4 mice/group). Scale bar = 100 μm. *p < 0.05, **p < 0.01, ***p < 0.001.

After confirming that erlotinib affects cognitive function, tau pathology, and astrogliosis in 3-month-old and 6-month-old PS19 mice, we examined the impact of erlotinib on cognitive function in another model of AD, 5xFAD mice, which overexpress Aβ. Prior to performing behavioral tests, the effect of erlotinib on EGFR phosphorylation, an on-target of erlotinib, was assessed. For this experiment, 3.5-month-old 5xFAD mice were treated with vehicle (5% DMSO + 10% PEG + 20% Tween80 + 65% D.W.) or erlotinib (20 mg/kg, i.p.) daily for 14 days, and immunofluorescence staining of brain slices was conducted with an anti-p-EGFR antibody. Erlotinib treatment significantly suppressed EGFR phosphorylation in the cortex and hippocampus in 3.5-month-old 5xFAD mice (**Figure 7A**).

We then examined the effect of erlotinib on cognitive function in 3-month-old 5xFAD mice. Three-month-old 5xFAD mice were treated with vehicle (5% DMSO + 10% PEG + 20% Tween80 + 65% D.W.) or erlotinib (20 mg/kg, i.p.) daily for 21 days, and the Y-maze test, NORT, and PAT were conducted. Similar to the effects of erlotinib treatment in PS19 mice, treating 3-month-old 5xFAD mice with erlotinib significantly enhanced alternation triads in the Y-maze test but did not alter recognition memory in the NORT nor aversive memory in the PAT compared with vehicle treatment (**Figures 7B–D**).

To explore the possible underlying mechanisms by which erlotinib improves short-term spatial memory in Aβ-overexpressing AD mice, we investigated the effects of erlotinib treatment on dendritic spine formation in 5xFAD mice. Strikingly, erlotinib treatment significantly increased hippocampal AO and BS dendritic spine numbers compared with vehicle treatment in 3-month-old 5xFAD mice (**Figure 7E**). These data suggest that erlotinib improves hippocampal-dependent short-term spatial memory by promoting hippocampal dendritic spinogenesis in 3-month-old 5xFAD mice.

We then investigated whether erlotinib regulates Aβ plaque accumulation in 3.5-month-old 5xFAD mice. The mice were injected with vehicle (5% DMSO + 10% PEG + 20% Tween80 + 65% D.W.) or erlotinib daily for 14 days, and immunofluorescence staining of brain slices was conducted with an anti-6E10 antibody that detects residues 1–16 of Aβ. Importantly, erlotinib treatment significantly suppressed 6E10-positive Aβ plaque deposition in the cortex and hippocampus compared with vehicle treatment in 3.5-month-old 5xFAD mice (**Figure 7F**). To further confirm the effect of erlotinib on Aβ plaque deposition, immunofluorescence staining was conducted with an anti-4G8 antibody detecting residues 18–23 of Aβ. The results showed that erlotinib treatment significantly suppressed 4G8-positive Aβ plaque deposition in the cortex and hippocampus compared with vehicle treatment in 3.5-month-old 5xFAD mice (**Supplementary Figure 1**). Taken together, these data suggest that erlotinib ameliorates Aβ-mediated cognitive impairments and Aβ pathology in 3- to 3.5-month-old 5xFAD mice.

### Erlotinib downregulates tau hyperphosphorylation and tau kinases in 5xFAD mice

**Figure 8:**
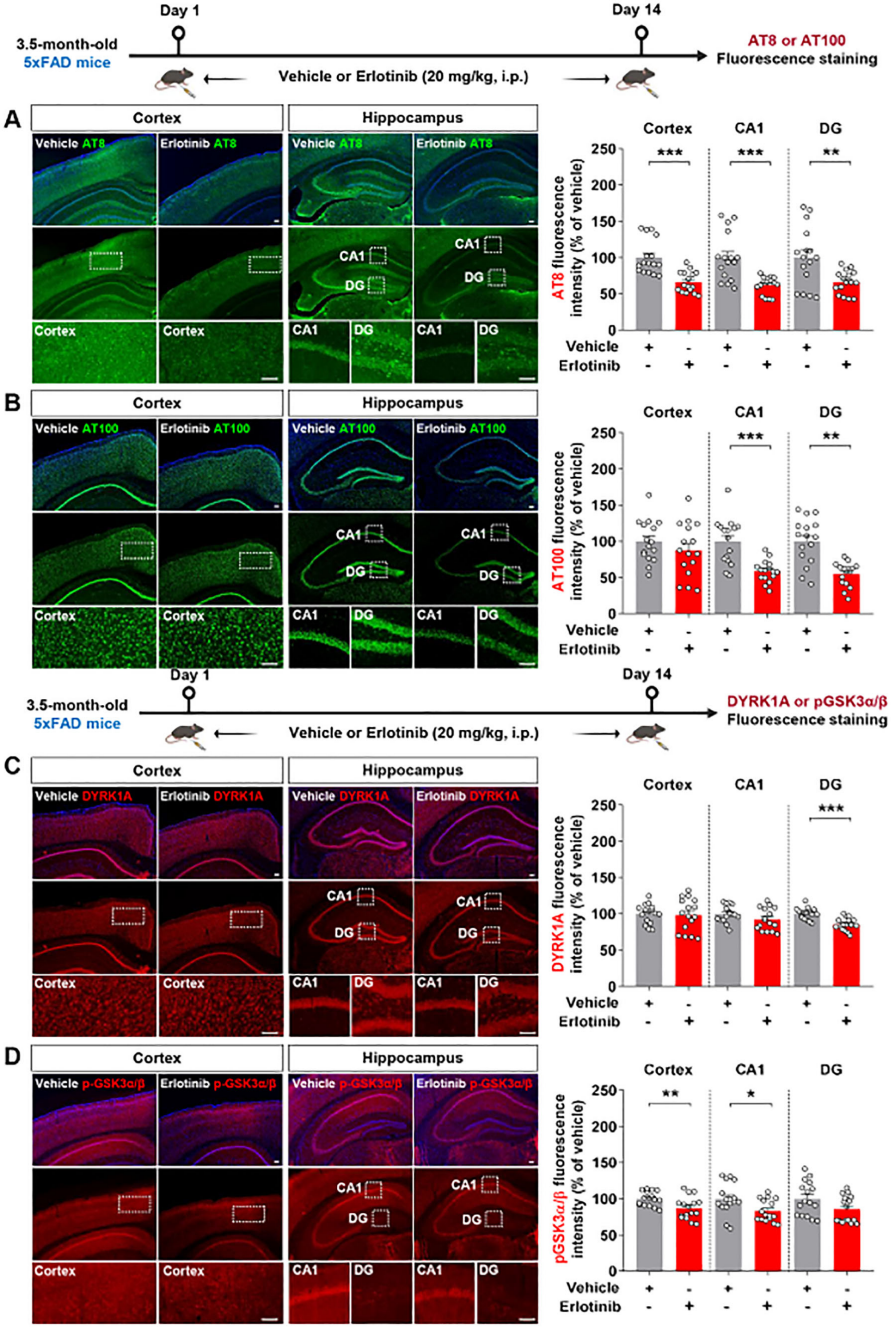
Erlotinib treatment decreases tau hyperphosphorylation and levels of the tau kinases DYRK1A and p-GSK3α/β in 5xFAD mice. **(A, B)** 3.5-month-old 5xFAD mice were injected with vehicle (5% DMSO + 10% PEG + 20% Tween80 + 65% D.W.) or erlotinib (20 mg/kg, i.p.) daily for 14 days, and brain slices were immunostained with anti-AT8 **(A)** and anti-AT100 **(B)** antibodies (n = 16 brain slices from 4 mice/group). **(C, D)** 3.5-month-old 5xFAD mice were injected with vehicle (5% DMSO + 10% PEG + 20% Tween80 + 65% D.W.) or erlotinib (20 mg/kg, i.p.) daily for 14 days, and brain slices were immunostained with anti-DYRK1A and anti-pGSK3α/β antibodies (n = 16 brain slices from 4 mice/group). Scale bar = 100 μm. *p < 0.05, **p < 0.01, ***p < 0.001.

Since erlotinib ameliorated tauopathy in PS19 mice, we examined the effects of erlotinib on tau hyperphosphorylation in 3.5-month-old 5xFAD mice. The mice were administered vehicle (5% DMSO + 10% PEG + 20% Tween80 + 65% D.W.) or erlotinib (20 mg/kg, i.p.) daily for 14 days, and immunofluorescence staining of brain slices was conducted with anti-AT8 and anti-AT100 antibodies. Erlotinib treatment significantly decreased tau hyperphosphorylation at Ser202/Thr205 (AT8) in the cortex and hippocampus in 3.5-month-old 5xFAD mice (**Figure 8A**). In addition, erlotinib treatment significantly reduced tau hyperphosphorylation at Thr212/Ser214 (AT100) in the hippocampus but not the cortex (**Figure 8B**).

We then investigated the mechanisms by which erlotinib reduces tau hyperphosphorylation by examining tau kinase levels/activity. To address this, 3.5-month-old 5xFAD mice were administered vehicle (5% DMSO + 10% PEG + 20% Tween80 + 65% D.W.) or erlotinib (20 mg/kg, i.p.) daily for 14 days, and immunofluorescence staining of brain slices was conducted with anti-DYRK1A and p-GSK3α/β antibodies. Erlotinib treatment significantly reduced DYRK1A expression in the hippocampal DG region and GSK3α/β phosphorylation in the cortex and hippocampal CA1 region in 3.5-month-old 5xFAD mice (**Figures 8C, D**). Collectively, these results indicate that erlotinib treatment reduces tau hyperphosphorylation and tau kinase levels/activity in this mouse model of the early phase of AD.

### Erlotinib downregulates reactive astrogliosis in 5xFAD mice and PACs from 5xFAD mice

To investigate whether erlotinib affects Aβ-induced microgliosis in 5xFAD mice, 3.5-month-old 5xFAD mice and wild-type (WT) mice were administered vehicle (5% DMSO + 10% PEG + 20% Tween80 + 65% D.W.) or erlotinib (20 mg/kg, i.p.) daily for 14 days, and immunofluorescence staining of brain slices was conducted with anti-Iba-1 and anti-GFAP antibodies. Iba-1 fluorescence intensity, Iba-1-positive cell number, and Iba-1-positive area were significantly greater in vehicle-treated 3.5-month-old 5xFAD mice than in vehicle-treated 3.5-month-old WT mice (**Supplementary Figure 2**). However, compared with the corresponding vehicle-treated mice, erlotinib treatment did not alter Iba-1 fluorescence intensity, Iba-1-positive cell number, or Iba-1-positive area in 3.5-month-old WT mice and 5xFAD mice (**Supplementary Figure 2**).

**Figure 9:**
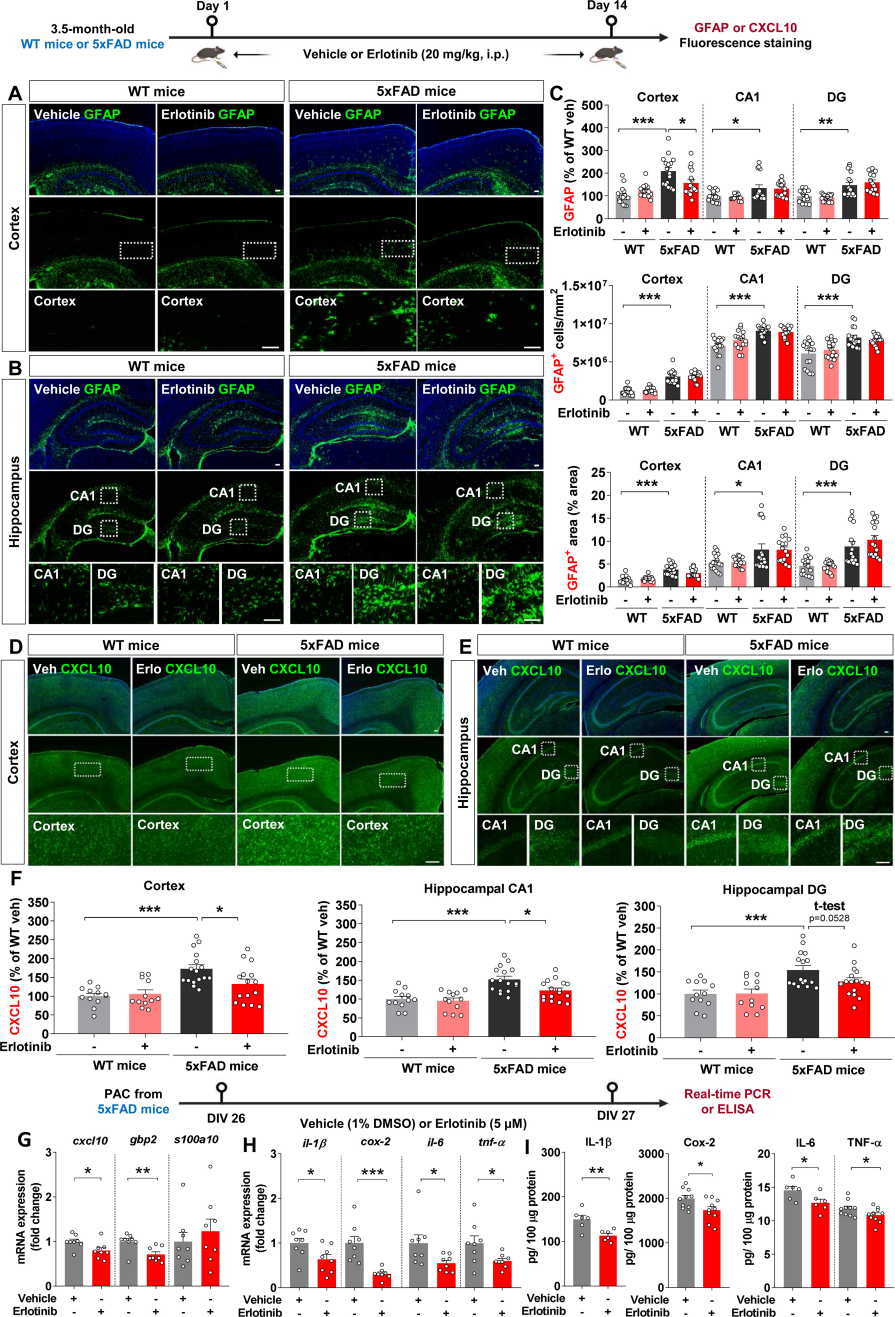
Erlotinib treatment significantly decreases reactive astrogliosis in 5xFAD mice and primary astrocytes (PACs) from 5xFAD mice. **(A–C)** 3.5-month-old WT and 5xFAD mice were injected with vehicle (5% DMSO + 10% PEG + 20% Tween80 + 65% D.W.) or erlotinib (20 mg/kg, i.p.) daily for 14 days, and immunofluorescence staining was conducted with an anti-GFAP antibody (n = 16 brain slices from 4 mice/group). **(D–F)** 3.5-month-old WT and 5xFAD mice were injected with vehicle (5% DMSO + 10% PEG + 20% Tween80 + 65% D.W.) or erlotinib (20 mg/kg, i.p.) daily for 14 days, and immunofluorescence staining was conducted with an anti-CXCL10 antibody (n = 12-16 brain slices from 3-4 mice/group). **(G, H)** PACs from 5xFAD mice were treated with vehicle (1% DMSO) or erlotinib (5 μM) for 24 h, and the relative mRNA levels of the indicated genes were analyzed by real-time PCR (n = 8/group). **(I)** PACs from 5xFAD mice were treated with vehicle (1% DMSO) or erlotinib (5 μM) for 24 h, and protein levels of proinflammatory cytokines were analyzed by ELISA (n = 6-10/group). Scale bar = 100 μm. *p<0.05, **p < 0.01, ***p < 0.001.

We then assessed the effects of erlotinib on Aβ-mediated astrogliosis in 5xFAD mice. GFAP fluorescence intensity, GFAP-positive cell number, and GFAP-positive area were significantly greater in vehicle-treated 3.5-month-old 5xFAD mice than in vehicle-treated WT mice (**Figures 9A–C**). Interestingly, erlotinib treatment significantly decreased GFAP fluorescence intensity in the cortex but not in the hippocampus in 3.5-month-old 5xFAD mice (**Figures 9A–C**).

To examine whether erlotinib modulates the AD-associated reactive astrocyte population, 3.5-month-old 5xFAD mice and WT mice were administered vehicle (5% DMSO + 10% PEG + 20% Tween80 + 65% D.W.) or erlotinib (20 mg/kg, i.p.) daily for 14 days, and immunofluorescence staining of brain slices was conducted with an anti-CXCL10 antibody to detect reactive astrocytes. Importantly, erlotinib treatment significantly decreased CXCL10 fluorescence intensity in the cortex and hippocampus in 3.5-month-old 5xFAD mice (**Figures 9D–F**). These data indicate that erlotinib treatment mitigates Aβ-induced astrogliosis but not Aβ-mediated microgliosis in 3.5-month-old 5xFAD mice.

We then analyzed the effect of erlotinib on the reactive state of astrocytes *in vitro*. PACs from 5xFAD mice were treated with vehicle (1% DMSO) or erlotinib (5 μM) for 24 h. Real-time PCR analysis showed that erlotinib treatment significantly decreased *cxcl10* (a marker of reactive astrocytes) and *gbp2* (a marker of proinflammatory A1 astrocytes) mRNA levels without altering *s100a10* (a marker of anti-inflammatory A2 astrocytes) mRNA levels (**Figure 9G**). In parallel with these results, erlotinib treatment significantly downregulated proinflammatory cytokine *il-1β*, *cox-2*, *il-6*, and *tnf-α* mRNA levels in PACs from 5xFAD mice (**Figure 9H**).

Finally, to examine whether erlotinib alters protein levels of proinflammatory cytokines in PACs from 5xFAD mice, PACs were treated with vehicle (1% DMSO) or erlotinib (5 μM) for 24 h, and ELISA was conducted. Erlotinib treatment significantly decreased proinflammatory cytokine IL-1β, COX-2, IL-6, and TNF-α protein levels in PACs from 5xFAD mice (**Figure 9I**). Taken together, these data suggest that erlotinib suppresses reactive astrogliosis and proinflammatory cytokine levels in this mouse model of the early phase of AD and/or in PACs from these mice.

## Discussion

In this study, we demonstrated for the first time that erlotinib enhances cognitive function and hippocampal dendritic spinogenesis in 6-month-old PS19 mice, a mouse model of AD. Strikingly, treating 3-month-old and 6-month-old PS19 mice with erlotinib attenuated tau hyperphosphorylation (Ser202/Thr205 or Thr231) and further aggregation of tau into PHFs (Ser396 and Ser404) and NFTs by inhibiting tau kinases. In addition, erlotinib treatment downregulated astrocytic activation and proinflammatory cytokine levels in 6-month-old PS19 mice or PACs from PS19 mice. In 3- to 3.5-month-old 5xFAD mice, erlotinib improved short-term memory and dendritic spine formation and reduced the number of Aβ plaques and tau phosphorylation. Moreover, erlotinib ameliorated reactive astrogliosis and neuroinflammatory responses associated with AD pathophysiology in 3.5-month-old 5xFAD mice and PACs from 5xFAD mice. These findings suggest that erlotinib is a potent multitarget (Aβ/tau/memory/neuroinflammation-linked) therapeutic for AD.

EGFR is expressed in the cortex and hippocampus, which leads to increased levels of β-tubulin protein, neurofilaments and the axon protein tau in the adult mammalian brain (24, 25). A recent study found that hippocampal *egfr* mRNA levels are significantly higher in 9-month-old PS19 mice than in WT littermates (26). Another study demonstrated that the pEGFR/EGFR ratio is increased in 8-month-old APP/PS1 double Tg mice, whereas the EGFR/β-actin ratio and pEGFR/β-actin ratio are decreased (9). A study of patients with AD showed that *egfr* mRNA and EGFR protein levels are significantly higher in those at Braak stages V-VI (fully developed AD) than in those at Braak stages 0-II (clinically silent) (27). These reports suggest that EGFR is a pivotal target for AD and that EGFR and pEGFR levels/activity in the brain should be considered before administering EGFR inhibitors.

In the present study, we found that erlotinib treatment significantly decreased p-EGFR expression in 3-month-old and 6-month-old PS19 mice as well as in 3.5-month-old 5xFAD mice (**Figures 1**, **8**). Interestingly, treating 3-month-old PS19 mice with erlotinib significantly suppressed p-EGFR expression in the cortex and hippocampus (**Figure 1A**). Moreover, erlotinib treatment significantly decreased EGFR phosphorylation in the cortex and hippocampal DG region but not in the CA1 region in 6-month-old PS19 mice (**Figure 1B**). These results raise the following question: Why do the effects of erlotinib on the expression of its target (p-EGFR) differ between regions of the hippocampus? One possibility is that EGFR is differentially expressed in hippocampal subregions (DG, CA1, and CA3), resulting in different inhibitory effects of erlotinib on its target (EGFR) across these subregions. We will further examine hippocampal subregional EGFR expression in PS19 mice in future studies.

In APP/PS1 double Tg mice, the EGFR antagonist gefitinib improves memory impairments (9). However, whether the EGFR inhibitor erlotinib regulates learning and memory in PS19 mice and its mechanisms of action are unknown. Our investigation of the effects of erlotinib on tau-mediated deficits in cognitive function and dendritic spinogenesis in 6-month-old PS19 mice showed that erlotinib treatment improved short-term spatial memory and hippocampal dendritic spine formation (**Figure 1**). Our findings demonstrate for the first time that EGFR inhibition by erlotinib alleviates tau-mediated cognitive dysfunction by promoting hippocampal dendritic spine formation in 6-month-old PS19 mice.

Abnormal aggregation of the microtubule binding protein tau begins in the entorhinal cortex and then disseminates into the hippocampus. The EGFR subunit ErbB4 and p-tau^Ser202^ are overexpressed and colocalized in progressive supranuclear palsy (PSP), a form of dementia (28). We recently demonstrated that the EGFR inhibitor varlitinib suppresses p-tau^Thr212/Ser214^ and p-tau^Thr231^ levels in 3- and 6-month-old PS19 mice (29). However, the involvement of EGFR in tau-related pathoprogression has not been extensively investigated. We found that erlotinib significantly downregulated hyperphosphorylation of tau at Ser202/Thr205 and/or Thr231 in 3-month-old and 6-month-old PS19 mice (**Figures 2**, **3**).

PHFs are a major component of NFTs. Tau hyperphosphorylation initiates tau aggregation and formation of the filamentous form (PHF-tau), which is the predominant form of tau in AD patients (30). Among the tau epitopes associated with PHF phosphorylation, histological staining and immunoblot analysis of PHFs have shown that p-PHF^Ser309^ and p-PHF^Ser404^ are predominantly increased in the entorhinal cortex and hippocampus in 6-month-old PS19 mice (31). However, there is no *in vivo* or *in vitro* evidence that blockade or knockdown of EGFR influences PHF formation. Here, we found that erlotinib treatment significantly reduced p-PHF^Ser396^/p-PHF^Ser404^ and NFT levels (**Figure 4**). Interestingly, the levels of middle- and high-molecular-weight (>30 kDa) soluble p-PHF^Ser396^/p-PHF^Ser404^ and insoluble p-PHF^Ser396^ were reduced by erlotinib treatment (**Figure 4**), indicating that erlotinib induces dephosphorylation of oligomeric fibrillar tau proteins more effectively than it induces dephosphorylation of tau fragments. Taken together, these data indicate that erlotinib has the potential to reduce p-PHF levels, which may be required to decrease NFTs, the dominant pathology in 6-month-old PS19 mice.

Hyperphosphorylation and misfolding of tau protein are closely associated with tau kinase activity in mouse models of AD. We previously showed that the EGFR inhibitor varlitinib significantly downregulates DYRK1A levels in PS19 mice (29). Interestingly, a recent study found that transfection of U251 cells with DYRK1A siRNA reduces EGFR expression in a time-dependent manner (32). Given the involvement of DYRK1A in AD pathoprogression, it is possible that EGFR affects DYRK1A levels/activity and *vice versa* in PS19 mice. Importantly, we found that erlotinib inhibited DYRK1A levels in 3-and 6-month-old PS19 mice (**Figure 5**). Taken together with the literature, our findings raise the possibility that the inhibition of DYRK1A levels by erlotinib affects EGFR functions according to a feedforward loop in the brain. To evaluate this possibility, we further investigated the effects of erlotinib treatment on other tau kinases and found that erlotinib treatment diminished cortical p-GSK3α/β and p-CDK5 levels in 6-month-old PS19 mice (**Figure 5**). These data indicate that erlotinib reduces tau pathology by modulating DYRK1A, p-CDK5 and p-GSK3α/β levels in 6-month-old PS19 mice. Our previous and current results raise the following question: Given that EGFR or its ligand EGF activates the phosphorylation of various tau kinases, including DYRK1A (33, 34), why does varlitinib suppress only DYRK1A without altering pCDK5 and pGSK3α/β in PS19 mice, whereas erlotinib inhibits the expression of DYRK1A, pCDK5, and pGSK3α/β? The IC_50_ of erlotinib against MCF 10A cells with EGFR mutations (L858R) is 40 times higher than the IC_50_ of varlitinib (35), suggesting that the inhibitory effects of erlotinib on EGFR and tau kinase levels are stronger than those of varlitinib in PS19 mice. Further studies will reveal the specific underlying mechanisms by which erlotinib treatment reduces tau hyperphosphorylation by each tau kinase in PS19 mice.

EGFR is involved in the initiation of inflammatory signaling pathways *in vitro* and *in vivo* (36). In cortical astrocytic cultures, inhibiting EGFR attenuates oxygen and glucose deprivation-induced neuroinflammation (37). In addition, we reported that the EGFR inhibitor varlitinib attenuates LPS-evoked and tau-mediated glial activation in WT mice and PS19 mice, respectively (29). In the present study, we found that the EGFR inhibitor erlotinib attenuated astroglial activation but not microgliosis in 3.5-month-old PS19 mice and reduced proinflammatory cytokine *il-1β* and *cox-2* mRNA levels in PACs from PS19 mice (**Figure 9**). Why does erlotinib predominantly suppress astrocytic activation instead of microglial activation in 6-month-old PS19 mice? It is possible that EGFR inhibition modulates tau-mediated glial activation in an age-dependent manner. We previously reported that the EGFR inhibitor varlitinib downregulates astroglial/microglial activation in 3-month-old PS19 mice but significantly suppresses only astrogliosis in 6-month-old PS19 mice (29). Therefore, it is possible that EGFR inhibition sufficiently downregulates both microglial and astroglial activation in the early phase of tau pathology (3-month-old PS19 mice) but only partially suppresses astrogliosis and/or microgliosis in the intermediate phase (6-month-old PS19 mice). We will further address this issue in a future study.

AD-associated microglial/astroglial activation in the early stages of AD (3- to 4-month-old 5xFAD mice) is controversial. Specifically, Forner et al. reported that the numbers of Iba-1- and GFAP-positive cells did not differ between 4-month-old 5xFAD mice and WT mice (38). By contrast, Liu et al. reported that Iba-1 and GFAP expression and the numbers of Iba-1- and GFAP-positive cells were higher in 4-month-old 5xFAD mice than in WT mice (39). In the present study, we found that Iba-1 and GFAP immunostaining intensity and positive area/cell were significantly higher in 3.5-month-old 5xFAD mice than in WT mice (**Figure 9**; **Supplementary Figure 2**). Interestingly, erlotinib treatment alleviated Aβ-mediated astrogliosis without altering microglial activation in 3.5-month-old 5xFAD mice (**Figure 9**; **Supplementary Figure 2**), consistent with the effects of erlotinib treatment in 6-month-old PS19 mice (**Figure 6**). Since AD-associated microgliosis and astrogliosis are exaggerated in the late phase of AD, a future study will examine whether erlotinib differentially regulates AD-mediated microglial/astroglial activation in aged 5xFAD mice.

A limitation of the present study is that we did not investigate protein levels/activity of EGFR and p-EGFR in aged AD mouse models compared to their WT littermates. In addition, we did not examine whether erlotinib improves cognitive function or alleviates AD pathologies, including Aβ plaque burden, tauopathy, and AD-associated neuroinflammatory responses, in 8-month-old or 12-month-old AD mouse models. Future work will address these issues; if erlotinib does exhibit therapeutic effects in aged AD mouse models, then we will determine whether erlotinib ameliorates AD pathologies in an EGFR-dependent manner by using an AAV-EGFR-shRNA vector system.

## Conclusion

This study demonstrates that erlotinib ameliorates the P301S mutant form of human tau-mediated cognitive impairments by improving dendritic spine formation in mouse models of AD. In addition, this study is the first to show that erlotinib diminishes tau hyperphosphorylation and further aggregation into PHFs/NFTs by inhibiting the tau kinases DYRK1A, pGSK3α/β, and pCDK5 in 3-month-old and/or 6-month-old P301S PS19 mice. Erlotinib treatment ameliorates astrogliosis in PS19 mice and proinflammatory cytokine *il-1β* and *cox-2* mRNA levels in PACs from PS19 mice. In 3- to 3.5-month-old 5xFAD mice, erlotinib significantly improves spatial memory by promoting dendritic spinogenesis and reducing Aβ plaque deposition and tau hyperphosphorylation. Furthermore, erlotinib ameliorates reactive astrogliosis and proinflammatory responses in 5xFAD mice or PACs from 5xFAD mice. Overall, our results suggest that erlotinib is a potential multitarget therapeutic for AD.

## Data Availability

The original contributions presented in the study are included in the article/**Supplementary Material**. Further inquiries can be directed to the corresponding author.
