# Supplementary material for: Erlotinib regulates short-term memory, tau/Aβ pathology, and astrogliosis in mouse models of AD
Source: Front Immunol. 2024 Oct 7;15:1421455. doi: 10.3389/fimmu.2024.1421455 (PMC11491340; doi:10.3389/fimmu.2024.1421455)
Supplement: Supplementary file 1 [file DataSheet1.doc]

**Erlotinib regulates** **short-term memory, tau/Aβ pathology, and astrogliosis in mouse models of AD**

**Hyun-ju Lee ^1,2,✝^| Jeong-Woo Hwang^1,2,✝^| Jieun Kim^1^| A-Ran Jo^1,2^| Jin-Hee Park^1^| Yoo Joo Jeong^1,2,3^| Ji-Yeong Jang^1,2,3^| Su-Jeong Kim^1^| Jeong-Heon Song^2^| Hyang-Sook Hoe^1,2,3,*^**

^1^Department of Neural Development and Disease, Korea Brain Research Institute (KBRI), 61, Cheomdan-ro, Daegu, Republic of Korea; ^2^AI-based Neurodevelopmental Diseases Digital Therapeutics Group,Korea Brain Research Institute (KBRI), 61, Cheomdan-ro, Daegu, Republic of Korea; ^3^Department of Brain and Cognitive Sciences, Daegu Gyeongbuk Institute of Science & Technology, Daegu 42988, Republic of Korea.^✝^ These authors contributed equally to this work.

***Correspondence**

**Hyang-Sook Hoe, Ph.D.,** Department of Neural Development and Disease, AI-based Neurodevelopmental Diseases Digital Therapeutics Group, Korea Brain Research Institute (KBRI), 61, Cheomdan-ro, Dong-gu, Daegu, Republic of Korea; E-mail: sookhoe72@kbri.re.kr

**E-mail addresses of authors**

Hyun-ju Lee: hjlee@kbri.re.kr

Jeong-Woo Hwang: jmseil42@kbri.re.kr

Jieun Kim: [jieunkim@kbri.re.kr](mailto:jieunkim@kbri.re.kr)

A-Ran Jo: dkfks784@gmail.com

Jin-Hee Park: mingmeng1005@kbri.re.kr

Yoo Joo Jeong: yoojoo930@kbri.re.kr

Ji-Young Jang: [jangjy@kbri.re.kr](mailto:jangjy@kbri.re.kr)

Su-Jeong Kim:js03341@kbri.re.kr

Jeong-Heon Song: jhsong316@kbri.re.kr

Hyang-Sook Hoe: sookhoe72@kbri.re.kr

**
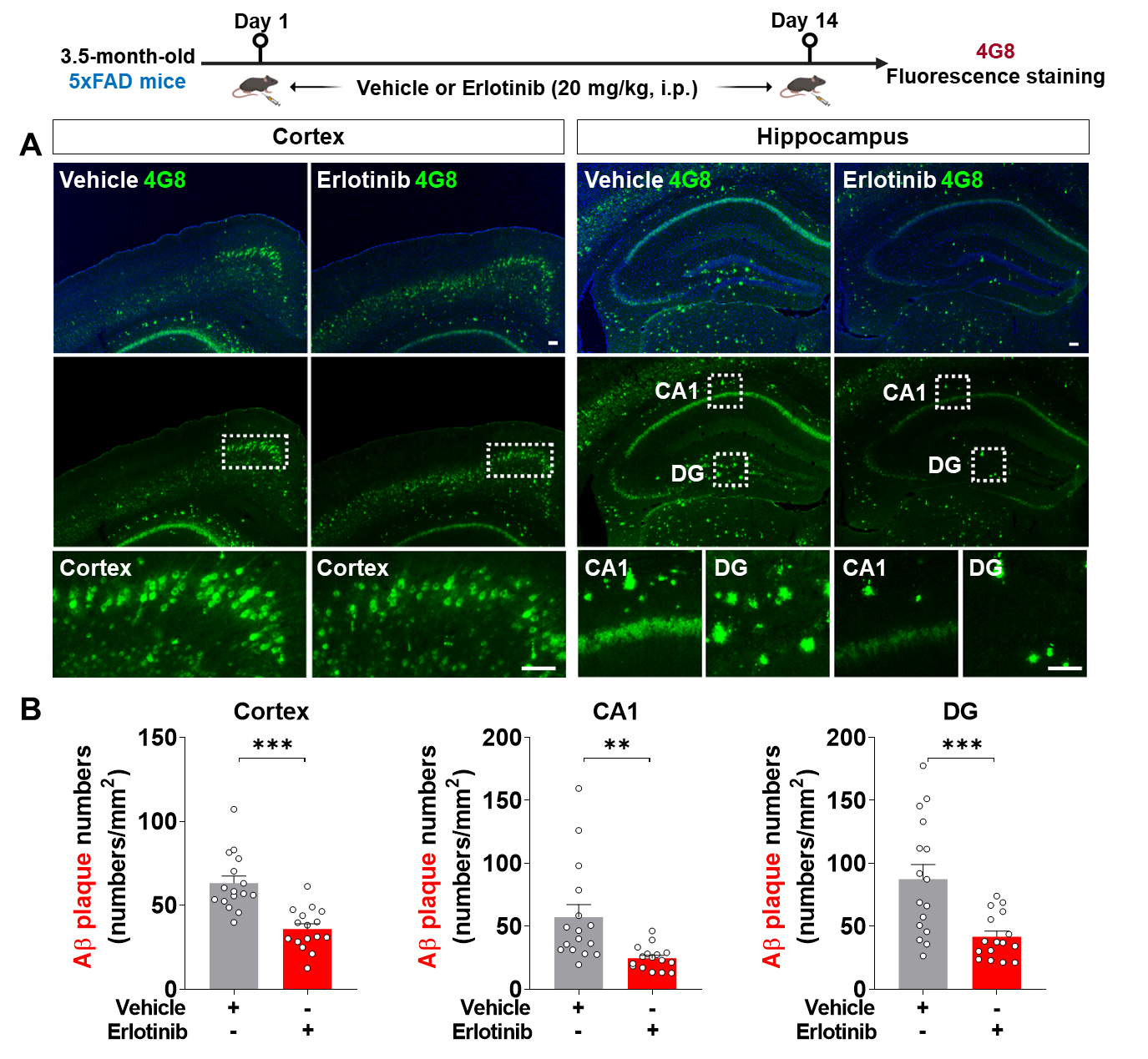
**

**Supplementary Figure 1**. Erlotinib decreases 4G8-positive Aβ plaque number in 5xFAD mice. **(A-B)** 3.5-month-old 5xFAD mice were injected with vehicle (5% DMSO + 10% PEG + 20% Tween80 + 65% D.W) or erlotinib (20 mg/kg, i.p.) daily for 14 days, and immunofluorescence staining was performed with anti-4G8 antibody (n = 16 brain slices from 4 mice/group). Scale bar = 100 μm. **p < 0.01, ***p < 0.001.

**
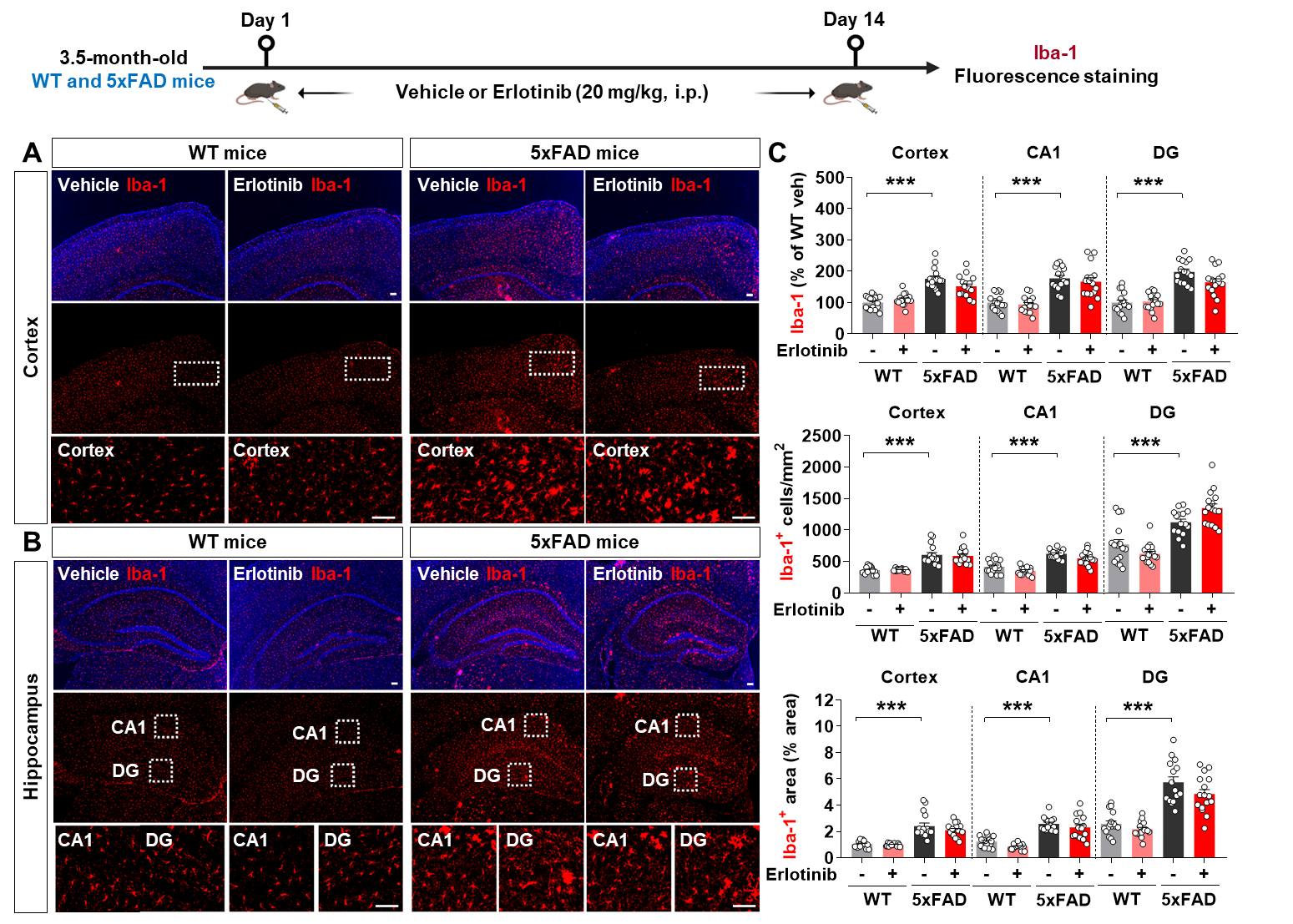
**

**Supplementary Figure 2. Erlotinib treatment did not alter microgliosis in WT mice and 5xFAD mice.** (A-C) 3.5-month-old WT and 5xFAD mice were injected with vehicle (5% DMSO + 10% PEG + 20% Tween80 + 65% D.W) or erlotinib (20 mg/kg, i.p.) daily for 14 days, and immunofluorescence staining was conducted with anti-Iba-1 antibody (n = 15-16 brain slices from 4 mice/group). Scale bar = 100 μm. ***p < 0.001.

**Additional file 1: Table S1.** Details of the statistical analyses in the present study

| **Figure 1A. pEGFR intensity in Entorhinal cortex of 3M** PS19 mice |
| --- |
| | Table Analyzed | p-EGFR | | --- | --- | |  |  | | Column B | - | | vs. | vs. | | Column A | + | |  |  | | Unpaired t test |  | | P value | 0.0351 | | P value summary | * | | Significantly different (P < 0.05)? | Yes | | One- or two-tailed P value? | Two-tailed | | t, df | t=2.207, df=30 | |
| **Figure 1A. pEGFR intensity in hippocampal CA1 of 3M** PS19 mice |
| | Table Analyzed | p-EGFR | | --- | --- | |  |  | | Column D | - | | vs. | vs. | | Column C | + | |  |  | | Unpaired t test |  | | P value | <0.0001 | | P value summary | **** | | Significantly different (P < 0.05)? | Yes | | One- or two-tailed P value? | Two-tailed | | t, df | t=5.635, df=30 | |
| **Figure 1A. pEGFR intensity in hippocampal DG of 3M** PS19 mice |
| | Table Analyzed | p-EGFR | | --- | --- | |  |  | | Column F | - | | vs. | vs. | | Column E | + | |  |  | | Unpaired t test |  | | P value | <0.0001 | | P value summary | **** | | Significantly different (P < 0.05)? | Yes | | One- or two-tailed P value? | Two-tailed | | t, df | t=5.630, df=30 | |
| **Figure 1B. pEGFR intensity in Entorhinal cortex of 6M** PS19 mice |
| | | Table Analyzed | p-EGFR | | --- | --- | |  |  | | Column B | - | | vs. | vs. | | Column A | + | |  |  | | Unpaired t test |  | | P value | 0.0005 | | P value summary | *** | | Significantly different (P < 0.05)? | Yes | | One- or two-tailed P value? | Two-tailed | | t, df | t=3.884, df=30 | |  | | --- | --- | --- | --- | --- | --- | --- | --- | --- | --- | --- | --- | --- | --- | --- | --- | --- | --- | --- | --- | --- | --- | --- | --- | --- | --- | |
| **Figure 1B. pEGFR intensity in Hippocampal CA1 of 6M** PS19 mice |
| | Table Analyzed | p-EGFR | | --- | --- | |  |  | | Column D | - | | vs. | vs. | | Column C | + | |  |  | | Unpaired t test |  | | P value | 0.1226 | | P value summary | ns | | Significantly different (P < 0.05)? | No | | One- or two-tailed P value? | Two-tailed | | t, df | t=1.589, df=30 | |
| **Figure 1B. pEGFR intensity in Hippocampal DG of 6M** PS19 mice |
| | | Table Analyzed | p-EGFR | | --- | --- | |  |  | | Column F | - | | vs. | vs. | | Column E | + | |  |  | | Unpaired t test |  | | P value | 0.0415 | | P value summary | * | | Significantly different (P < 0.05)? | Yes | | One- or two-tailed P value? | Two-tailed | | t, df | t=2.129, df=30 | |  | | --- | --- | --- | --- | --- | --- | --- | --- | --- | --- | --- | --- | --- | --- | --- | --- | --- | --- | --- | --- | --- | --- | --- | --- | --- | --- | |
| **Figure 1C. Alternation % in Y maze (6M PS19 mice)** |
| | Table Analyzed | Y-maze | | --- | --- | |  |  | | Column B | - | | vs. | vs. | | Column A | + | |  |  | | Unpaired t test with Welch's correction |  | | P value | 0.0412 | | P value summary | * | | Significantly different (P < 0.05)? | Yes | | One- or two-tailed P value? | One-tailed | | Welch-corrected t, df | t=1.838, df=18.43 | |
| **Figure 1C. Total arm entry numbers in Y maze (6M PS19 mice)** |
| | Table Analyzed | Y-maze | | --- | --- | |  |  | | Column B | - | | vs. | vs. | | Column A | + | |  |  | | Unpaired t test with Welch's correction |  | | P value | 0.4184 | | P value summary | ns | | Significantly different (P < 0.05)? | No | | One- or two-tailed P value? | One-tailed | | Welch-corrected t, df | t=0.2089, df=18.99 | |
| **Figure 1D. Object preference in NOR training (6M PS19 mice**) |
| | Table Analyzed | NORT | | --- | --- | |  |  | | Column B | Data Set-B | | vs. | vs. | | Column A | Data Set-A | |  |  | | Unpaired t test with Welch's correction |  | | P value | 0.9183 | | P value summary | ns | | Significantly different (P < 0.05)? | No | | One- or two-tailed P value? | Two-tailed | | Welch-corrected t, df | t=0.1040, df=18.97 | |
| **Figure 1D. Object preference in NOR test (6M PS19 mice**) |
| | Table Analyzed | NORT | | --- | --- | |  |  | | Column E | Data Set-E | | vs. | vs. | | Column D | Data Set-D | |  |  | | Unpaired t test with Welch's correction |  | | P value | 0.7071 | | P value summary | ns | | Significantly different (P < 0.05)? | No | | One- or two-tailed P value? | Two-tailed | | Welch-corrected t, df | t=0.3819, df=17.73 | |
| **Figure 1E. Escape latency in PAT (6M PS19 mice**) |
| | Table Analyzed | PAT |  |  |  | | --- | --- | --- | --- | --- | |  |  |  |  |  | | Mixed-effects model (REML) | Matching: Stacked |  |  |  | | Assume sphericity? | Yes |  |  |  | | Alpha | 0.05 |  |  |  | |  |  |  |  |  | | Fixed effects (type III) | P value | P value summary | Statistically significant (P < 0.05)? | F (DFn, DFd) | | Time | <0.0001 | **** | Yes | F (1, 19) = 29.37 | | Column Factor | 0.2425 | ns | No | F (1, 19) = 1.455 | | Time x Column Factor | 0.1174 | ns | No | F (1, 19) = 2.691 |  | Compare each cell mean with the other cell mean in that column |  |  |  |  |  | | --- | --- | --- | --- | --- | --- | |  |  |  |  |  |  | | Number of families | 1 |  |  |  |  | | Number of comparisons per family | 2 |  |  |  |  | | Alpha | 0.05 |  |  |  |  | |  |  |  |  |  |  | | Bonferroni's multiple comparisons test | Predicted (LS) mean diff. | 95.00% CI of diff. | Significant? | Summary | Adjusted P Value | |  |  |  |  |  |  | | training day - test day |  |  |  |  |  | | Vehicle | -48.92 | -94.52 to -3.319 | Yes | * | 0.0344 | | Erlotinib | -91.39 | -134.9 to -47.91 | Yes | *** | 0.0001 | |  |  |  |  |  |  | |
| **Figure 1F. Dendritic spine numbers in AO of Hippocampal CA1 from 6M PS19 mice** |
| | Table Analyzed | spine # | | --- | --- | |  |  | | Column B | Data Set-B | | vs. | vs. | | Column A | Data Set-A | |  |  | | Unpaired t test |  | | P value | 0.0088 | | P value summary | ** | | Significantly different (P < 0.05)? | Yes | | One- or two-tailed P value? | Two-tailed | | t, df | t=2.814, df=28 | |
| **Figure 1F. Dendritic spine numbers in BS of Hippocampal CA1 from 6M PS19 mice** |
| | Table Analyzed | spine # | | --- | --- | |  |  | | Column E | Data Set-E | | vs. | vs. | | Column D | Data Set-D | |  |  | | Unpaired t test |  | | P value | 0.0456 | | P value summary | * | | Significantly different (P < 0.05)? | Yes | | One- or two-tailed P value? | Two-tailed | | t, df | t=2.068, df=37 | |
| **Figure 2A. AT8 intensity in Entorhinal cortex of 3M** PS19 mice |
| | Table Analyzed | AT8 | | --- | --- | |  |  | | Column B | - | | vs. | vs. | | Column A | + | |  |  | | Unpaired t test |  | | P value | 0.0332 | | P value summary | * | | Significantly different (P < 0.05)? | Yes | | One- or two-tailed P value? | Two-tailed | | t, df | t=2.233, df=30 | |
| **Figure 2A. AT8 intensity in Hippocampal CA1 of 3M PS19 mice** |
| | Table Analyzed | AT8 | | --- | --- | |  |  | | Column D | - | | vs. | vs. | | Column C | + | |  |  | | Unpaired t test |  | | P value | 0.0255 | | P value summary | * | | Significantly different (P < 0.05)? | Yes | | One- or two-tailed P value? | Two-tailed | | t, df | t=2.351, df=30 | |
| **Figure 2A. AT8 intensity in Hippocampal DG of 3M PS19 mice** |
| | Table Analyzed | AT8 | | --- | --- | |  |  | | Column F | - | | vs. | vs. | | Column E | + | |  |  | | Unpaired t test |  | | P value | 0.0633 | | P value summary | ns | | Significantly different (P < 0.05)? | No | | One- or two-tailed P value? | Two-tailed | | t, df | t=1.928, df=30 | |
| **Figure 2B. AT100 intensity in Entorhinal cortex of 3M PS19 mice** |
| | Table Analyzed | AT100 | | --- | --- | |  |  | | Column B | - | | vs. | vs. | | Column A | + | |  |  | | Unpaired t test |  | | P value | 0.0005 | | P value summary | *** | | Significantly different (P < 0.05)? | Yes | | One- or two-tailed P value? | Two-tailed | | t, df | t=3.888, df=30 | |
| **Figure 2B. AT100 intensity in Hippocampal CA1 of 3M PS19 mice** |
| | Table Analyzed | AT100 | | --- | --- | |  |  | | Column D | - | | vs. | vs. | | Column C | + | |  |  | | Unpaired t test |  | | P value | 0.2349 | | P value summary | ns | | Significantly different (P < 0.05)? | No | | One- or two-tailed P value? | Two-tailed | | t, df | t=1.212, df=30 | |
| **Figure 2B. AT100 intensity in Hippocampal DG of 3M PS19 mice** |
| | Table Analyzed | AT100 | | --- | --- | |  |  | | Column F | - | | vs. | vs. | | Column E | + | |  |  | | Unpaired t test |  | | P value | 0.1219 | | P value summary | ns | | Significantly different (P < 0.05)? | No | | One- or two-tailed P value? | Two-tailed | | t, df | t=1.592, df=30 | |
| **Figure 2C. AT180 intensity in Entorhinal cortex of 3M PS19 mice** |
| | Table Analyzed | AT180 | | --- | --- | |  |  | | Column B | - | | vs. | vs. | | Column A | + | |  |  | | Unpaired t test |  | | P value | <0.0001 | | P value summary | **** | | Significantly different (P < 0.05)? | Yes | | One- or two-tailed P value? | Two-tailed | | t, df | t=4.979, df=25 | |
| **Figure 2C. AT180 intensity in Hippocampal CA1 of 3M PS19 mice** |
| | Table Analyzed | AT180 | | --- | --- | |  |  | | Column D | - | | vs. | vs. | | Column C | + | |  |  | | Unpaired t test |  | | P value | <0.0001 | | P value summary | **** | | Significantly different (P < 0.05)? | Yes | | One- or two-tailed P value? | Two-tailed | | t, df | t=4.974, df=26 | |
| **Figure 2C. AT180 intensity in Hippocampal DG of 3M PS19 mice** |
| | Table Analyzed | AT180 | | --- | --- | |  |  | | Column F | - | | vs. | vs. | | Column E | + | |  |  | | Unpaired t test |  | | P value | 0.0001 | | P value summary | *** | | Significantly different (P < 0.05)? | Yes | | One- or two-tailed P value? | Two-tailed | | t, df | t=4.472, df=26 | |  |  | |
| **Figure 3A. AT8 intensity in Entorhinal cortex of 6M PS19 mice** |
| | Table Analyzed | AT8 | | --- | --- | |  |  | | Column B | - | | vs. | vs. | | Column A | + | |  |  | | Unpaired t test |  | | P value | 0.9094 | | P value summary | ns | | Significantly different (P < 0.05)? | No | | One- or two-tailed P value? | Two-tailed | | t, df | t=0.1144, df=51 | |
| **Figure 3A. AT8 intensity in Hippocampal CA1 of 6M PS19 mice** |
| |  | | Table Analyzed | AT8 | | --- | --- | |  |  | | Column D | - | | vs. | vs. | | Column C | + | |  |  | | Unpaired t test |  | | P value | 0.0414 | | P value summary | * | | Significantly different (P < 0.05)? | Yes | | One- or two-tailed P value? | Two-tailed | | t, df | t=2.101, df=44 | | | --- | --- | --- | --- | --- | --- | --- | --- | --- | --- | --- | --- | --- | --- | --- | --- | --- | --- | --- | --- | --- | --- | --- | --- | --- | --- | |
| **Figure 3A. AT8 intensity in Hippocampal DG of 6M PS19 mice** |
| | | Table Analyzed | AT8 | | --- | --- | |  |  | | Column F | - | | vs. | vs. | | Column E | + | |  |  | | Unpaired t test |  | | P value | 0.0234 | | P value summary | * | | Significantly different (P < 0.05)? | Yes | | One- or two-tailed P value? | Two-tailed | | t, df | t=2.348, df=44 | |  | | --- | --- | --- | --- | --- | --- | --- | --- | --- | --- | --- | --- | --- | --- | --- | --- | --- | --- | --- | --- | --- | --- | --- | --- | --- | --- | |
| **Figure 3B. AT100 intensity in Entorhinal cortex of 6M PS19 mice** |
| | Table Analyzed | AT100 | | --- | --- | |  |  | | Column B | - | | vs. | vs. | | Column A | + | |  |  | | Unpaired t test |  | | P value | 0.1800 | | P value summary | ns | | Significantly different (P < 0.05)? | No | | One- or two-tailed P value? | Two-tailed | | t, df | t=1.361, df=47 | |
| **Figure 3B. AT100 intensity in Hippocampal CA1 of 6M PS19 mice** |
| |  | | Table Analyzed | AT100 | | --- | --- | |  |  | | Column D | - | | vs. | vs. | | Column C | + | |  |  | | Unpaired t test |  | | P value | 0.1558 | | P value summary | ns | | Significantly different (P < 0.05)? | No | | One- or two-tailed P value? | Two-tailed | | t, df | t=1.443, df=47 | | | --- | --- | --- | --- | --- | --- | --- | --- | --- | --- | --- | --- | --- | --- | --- | --- | --- | --- | --- | --- | --- | --- | --- | --- | --- | --- | |
| **Figure 3B. AT100 intensity in Hippocampal DG of 6M PS19 mice** |
| | Table Analyzed | AT100 | | --- | --- | |  |  | | Column F | - | | vs. | vs. | | Column E | + | |  |  | | Unpaired t test |  | | P value | >0.9999 | | P value summary | ns | | Significantly different (P < 0.05)? | No | | One- or two-tailed P value? | Two-tailed | | t, df | t=0.000, df=58 | |
| **Figure 3C. AT180 intensity in Entorhinal cortex of 6M PS19 mice** |
| | Table Analyzed | AT180 | | --- | --- | |  |  | | Column B | - | | vs. | vs. | | Column A | + | |  |  | | Unpaired t test |  | | P value | 0.0764 | | P value summary | ns | | Significantly different (P < 0.05)? | No | | One- or two-tailed P value? | Two-tailed | | t, df | t=1.814, df=45 | |
| **Figure 3C. AT180 intensity in Hippocampal CA1 of 6M PS19 mice** |
| |  | | Table Analyzed | AT180 | | --- | --- | |  |  | | Column D | - | | vs. | vs. | | Column C | + | |  |  | | Unpaired t test |  | | P value | 0.0681 | | P value summary | ns | | Significantly different (P < 0.05)? | No | | One- or two-tailed P value? | Two-tailed | | t, df | t=1.866, df=49 | | | --- | --- | --- | --- | --- | --- | --- | --- | --- | --- | --- | --- | --- | --- | --- | --- | --- | --- | --- | --- | --- | --- | --- | --- | --- | --- | |
| **Figure 3C. AT180 intensity in Hippocampal DG of 6M PS19 mice** |
| | Table Analyzed | AT180 | | --- | --- | |  |  | | Column F | - | | vs. | vs. | | Column E | + | |  |  | | Unpaired t test |  | | P value | 0.8071 | | P value summary | ns | | Significantly different (P < 0.05)? | No | | One- or two-tailed P value? | Two-tailed | | t, df | t=0.2456, df=48 | |
| **Figure 3D. AT8 expression in soluble fraction of Entorhinal cortex from 6M PS19 mice** |
| | Table Analyzed | AT8 | | --- | --- | |  |  | | Column B | - | | vs. | vs. | | Column A | + | |  |  | | Unpaired t test |  | | P value | 0.0315 | | P value summary | * | | Significantly different (P < 0.05)? | Yes | | One- or two-tailed P value? | One-tailed | | t, df | t=2.019, df=14 | |
| **Figure 3D. AT8 expression in IN-soluble fraction of Entorhinal cortex from 6M PS19 mice** |
| | Table Analyzed | AT8 | | --- | --- | |  |  | | Column D | - | | vs. | vs. | | Column C | + | |  |  | | Unpaired t test |  | | P value | 0.3846 | | P value summary | ns | | Significantly different (P < 0.05)? | No | | One- or two-tailed P value? | One-tailed | | t, df | t=0.2993, df=14 | |
| **Figure 3E. AT8 expression in soluble fraction of Hippocampus from 6M PS19 mice** |
| | Table Analyzed | AT8 | | --- | --- | |  |  | | Column B | - | | vs. | vs. | | Column A | + | |  |  | | Unpaired t test |  | | P value | 0.2396 | | P value summary | ns | | Significantly different (P < 0.05)? | No | | One- or two-tailed P value? | One-tailed | | t, df | t=0.7269, df=14 | |
| **Figure 3E. AT8 expression in IN-soluble fraction of Hippocampus from 6M PS19 mice** |
| | Table Analyzed | AT8 | | --- | --- | |  |  | | Column D | - | | vs. | vs. | | Column C | + | |  |  | | Unpaired t test |  | | P value | 0.0400 | | P value summary | * | | Significantly different (P < 0.05)? | Yes | | One- or two-tailed P value? | One-tailed | | t, df | t=1.887, df=14 | |
| **Figure 4A. pSer396 expression in soluble fraction (Tau) of Entorhinal cortex from 6M PS19 mice** |
| |  |  |  | | --- | --- | --- | | Table Analyzed | p-Ser396 EC | | |  |  | | | Column B | - + | | | vs. | vs. | | | Column A | + - | | |  |  | | | Unpaired t test |  | | | P value | 0.0032 | | | P value summary | ** | | | Significantly different (P < 0.05)? | Yes | | | One- or two-tailed P value? | Two-tailed | | | t, df | t=3.544, df=14 | | |
| **Figure 4A. pSer396 expression in soluble fraction (Fragments) of Entorhinal cortex from 6M PS19 mice** |
| | Table Analyzed | p-Ser396 EC | | --- | --- | |  |  | | Column D | - + | | vs. | vs. | | Column C | + - | |  |  | | Unpaired t test |  | | P value | 0.1930 | | P value summary | ns | | Significantly different (P < 0.05)? | No | | One- or two-tailed P value? | One-tailed | | t, df | t=0.8950, df=14 | |  |  | |
| **Figure 4B. pSer396 expression in IN-soluble fraction (Tau) of Entorhinal cortex from 6M PS19 mice** |
| | Table Analyzed | p-Ser396 EC | | --- | --- | |  |  | | Column B | - + | | vs. | vs. | | Column A | + - | |  |  | | Unpaired t test |  | | P value | 0.2854 | | P value summary | ns | | Significantly different (P < 0.05)? | No | | One- or two-tailed P value? | Two-tailed | | t, df | t=1.111, df=14 | |
| **Figure 4C. pSer396 expression in soluble fraction (Tau) of Hippocampus from 6M PS19 mice** |
| | Table Analyzed | | p-Ser396 HC |  | | --- | --- | |  |  | | Column B | - + | | vs. | vs. | | Column A | + - | |  |  | | Unpaired t test |  | | P value | 0.2135 | | P value summary | ns | | Significantly different (P < 0.05)? | No | | One- or two-tailed P value? | Two-tailed | | t, df | t=1.329, df=10 | | | --- | --- | --- | --- | --- | --- | --- | --- | --- | --- | --- | --- | --- | --- | --- | --- | --- | --- | --- | --- | --- | --- | --- | --- | --- | --- | |  |  | |
| **Figure 4C. pSer396 expression in soluble fraction (Fragments) of Hippocampus from 6M PS19 mice** |
| | Table Analyzed | p-Ser396 HC | | --- | --- | |  |  | | Column D | - + | | vs. | vs. | | Column C | + - | |  |  | | Unpaired t test |  | | P value | 0.2546 | | P value summary | ns | | Significantly different (P < 0.05)? | No | | One- or two-tailed P value? | Two-tailed | | t, df | t=1.209, df=10 | |
| **Figure 4D. pSer396 expression in IN-soluble fraction (Tau) of Hippocampus from 6M PS19 mice** |
| | Table Analyzed | p-Ser396 EC | | --- | --- | |  |  | | Column B | - + | | vs. | vs. | | Column A | + - | |  |  | | Unpaired t test |  | | P value | 0.0075 | | P value summary | ** | | Significantly different (P < 0.05)? | Yes | | One- or two-tailed P value? | Two-tailed | | t, df | t=3.161, df=13 | |
| **Figure 4E. pSer404 expression in soluble fraction (Tau) of Entorhinal cortex from 6M PS19 mice** |
| | Table Analyzed | p-Ser404 EC | | --- | --- | |  |  | | Column B | - + | | vs. | vs. | | Column A | + - | |  |  | | Unpaired t test |  | | P value | 0.0259 | | P value summary | * | | Significantly different (P < 0.05)? | Yes | | One- or two-tailed P value? | One-tailed | | t, df | t=2.126, df=14 | |
| **Figure 4E. pSer404 expression in soluble fraction (Fragments) of Entorhinal cortex from 6M PS19 mice** |
| | Table Analyzed | p-Ser404 EC | | --- | --- | |  |  | | Column D | - + | | vs. | vs. | | Column C | + - | |  |  | | Unpaired t test |  | | P value | 0.0747 | | P value summary | ns | | Significantly different (P < 0.05)? | No | | One- or two-tailed P value? | One-tailed | | t, df | t=1.526, df=14 | |
| **Figure 4F. pSer404 expression in IN-soluble fraction (Tau) of Entorhinal cortex from 6M PS19 mice** |
| |  |  | | --- | --- | | Table Analyzed | p-Ser404 EC | |  |  | | Column B | - + | | vs. | vs. | | Column A | + - | |  |  | | Unpaired t test |  | | P value | 0.3029 | | P value summary | ns | | Significantly different (P < 0.05)? | No | | One- or two-tailed P value? | Two-tailed | | t, df | t=1.069, df=14 | |
| **Figure 4G. pSer404 expression in soluble fraction (Tau) of Hippocampus from 6M PS19 mice** |
| | Table Analyzed | p-Ser404 HC | | --- | --- | |  |  | | Column B | - + | | vs. | vs. | | Column A | + - | |  |  | | Unpaired t test |  | | P value | 0.3805 | | P value summary | ns | | Significantly different (P < 0.05)? | No | | One- or two-tailed P value? | Two-tailed | | t, df | t=0.9078, df=13 | |
| **Figure 4G. pSer404 expression in soluble fraction (Fragments) of Hippocampus from 6M PS19 mice** |
| | Table Analyzed | p-Ser404 HC | | --- | --- | |  |  | | Column D | - + | | vs. | vs. | | Column C | + - | |  |  | | Unpaired t test |  | | P value | 0.9271 | | P value summary | ns | | Significantly different (P < 0.05)? | No | | One- or two-tailed P value? | Two-tailed | | t, df | t=0.09322, df=14 | |
| **Figure 4H. pSer404 expression in IN-soluble fraction (Tau) of Hippocampus from 6M PS19 mice** |
| | Table Analyzed | p-Ser404 HC | | --- | --- | |  |  | | Column B | - + | | vs. | vs. | | Column A | + - | |  |  | | Unpaired t test |  | | P value | 0.9691 | | P value summary | ns | | Significantly different (P < 0.05)? | No | | One- or two-tailed P value? | Two-tailed | | t, df | t=0.03953, df=13 | |
| **Figure 4I. Tau5 expression in IN-soluble fraction (Tau) of Entorhinal cortex from 6M PS19 mice** |
| | Table Analyzed | Tau5 | | --- | --- | |  |  | | Column D | - | | vs. | vs. | | Column C | + | |  |  | | Unpaired t test |  | | P value | 0.3905 | | P value summary | ns | | Significantly different (P < 0.05)? | No | | One- or two-tailed P value? | Two-tailed | | t, df | t=0.8975, df=10 | |
| **Figure 4I. Tau5 expression in IN-soluble fraction (Tau) of Hippocampus from 6M PS19 mice** |
| | Table Analyzed | Tau5 | | --- | --- | |  |  | | Column D | - | | vs. | vs. | | Column C | + | |  |  | | Unpaired t test |  | | P value | 0.5588 | | P value summary | ns | | Significantly different (P < 0.05)? | No | | One- or two-tailed P value? | Two-tailed | | t, df | t=0.6048, df=10 | |
| **Figure 4J. NFT intensity in Entorhinal cortex of 6M** PS19 mice |
| | Table Analyzed | NFT | | --- | --- | |  |  | | Column B | - | | vs. | vs. | | Column A | + | |  |  | | Unpaired t test |  | | P value | 0.0003 | | P value summary | *** | | Significantly different (P < 0.05)? | Yes | | One- or two-tailed P value? | Two-tailed | | t, df | t=3.924, df=47 | |
| **Figure 4J. NFT intensity in Hippocampal CA1 of 6M** PS19 mice |
| | Table Analyzed | NFT - CA1 | | --- | --- | |  |  | | Column A | + | | vs. | vs. | | Column B | - | |  |  | | Unpaired t test |  | | P value | <0.0001 | | P value summary | **** | | Significantly different (P < 0.05)? | Yes | | One- or two-tailed P value? | Two-tailed | | t, df | t=4.364, df=50 | |
| **Figure 4J. NFT intensity in Hippocampal DG of 6M** PS19 mice |
| | Table Analyzed | NFT - dg | | --- | --- | |  |  | | Column A | + | | vs. | vs. | | Column B | - | |  |  | | Unpaired t test |  | | P value | <0.0001 | | P value summary | **** | | Significantly different (P < 0.05)? | Yes | | One- or two-tailed P value? | Two-tailed | | t, df | t=4.330, df=50 | |
| **Figure 5A. DYRK1A intensity in Entorhinal cortex of 3M PS19 mice** |
| | Table Analyzed | DYRK1A | | --- | --- | |  |  | | Column B | - | | vs. | vs. | | Column A | + | |  |  | | Unpaired t test |  | | P value | 0.0002 | | P value summary | *** | | Significantly different (P < 0.05)? | Yes | | One- or two-tailed P value? | Two-tailed | | t, df | t=4.254, df=30 | |
| **Figure 5A. DYRK1A intensity in Hippocampal CA1 of 3M PS19 mice** |
| | Table Analyzed | DYRK1A | | --- | --- | |  |  | | Column D | - + | | vs. | vs. | | Column C | + - | |  |  | | Unpaired t test |  | | P value | 0.0128 | | P value summary | * | | Significantly different (P < 0.05)? | Yes | | One- or two-tailed P value? | One-tailed | | t, df | t=2.350, df=30 | |
| **Figure 5A. DYRK1A intensity in Hippocampal DG of 3M PS19 mice** |
| | Table Analyzed | DYRK1A | | --- | --- | |  |  | | Column F | - | | vs. | vs. | | Column E | + | |  |  | | Unpaired t test |  | | P value | 0.1550 | | P value summary | ns | | Significantly different (P < 0.05)? | No | | One- or two-tailed P value? | Two-tailed | | t, df | t=1.459, df=30 | |
| **Figure 5B. DYRK1A expression in Entorhinal cortex of 6M PS19 mice** |
| | Table Analyzed | DYRK1A | | --- | --- | |  |  | | Column B | - | | vs. | vs. | | Column A | + | |  |  | | Paired t test |  | | P value | 0.0036 | | P value summary | ** | | Significantly different (P < 0.05)? | Yes | | One- or two-tailed P value? | Two-tailed | | t, df | t=4.283, df=7 | | Number of pairs | 8 | |
| **Figure 5C. DYRK1A expression in Hippocampus of 6M PS19 mice** |
| | Table Analyzed | DYRK1A | | --- | --- | |  |  | | Column B | - | | vs. | vs. | | Column A | + | |  |  | | Paired t test |  | | P value | 0.3973 | | P value summary | ns | | Significantly different (P < 0.05)? | No | | One- or two-tailed P value? | Two-tailed | | t, df | t=0.9015, df=7 | | Number of pairs | 8 | |
| **Figure 5D. DYRK1A intensity in Entorhinal cortex of 6M PS19 mice** |
| | Table Analyzed | DYRK1A | | --- | --- | |  |  | | Column B | - + | | vs. | vs. | | Column A | + - | |  |  | | Unpaired t test |  | | P value | 0.0052 | | P value summary | ** | | Significantly different (P < 0.05)? | Yes | | One- or two-tailed P value? | Two-tailed | | t, df | t=2.929, df=48 | |
| **Figure 5D. DYRK1A intensity in Hippocampal CA1 of 6M PS19 mice** |
| | Table Analyzed | DYRK1A | | --- | --- | |  |  | | Column D | - + | | vs. | vs. | | Column C | + - | |  |  | | Unpaired t test |  | | P value | 0.0482 | | P value summary | * | | Significantly different (P < 0.05)? | Yes | | One- or two-tailed P value? | One-tailed | | t, df | t=1.696, df=47 | |
| **Figure 5D. DYRK1A intensity in Hippocampal DG of 6M PS19 mice** |
| | Table Analyzed | DYRK1A | | --- | --- | |  |  | | Column F | - + | | vs. | vs. | | Column E | + - | |  |  | | Unpaired t test |  | | P value | 0.2113 | | P value summary | ns | | Significantly different (P < 0.05)? | No | | One- or two-tailed P value? | Two-tailed | | t, df | t=1.267, df=47 | |
| **Figure 5E. pGSK3α/β expression in Entorhinal cortex of 6M PS19 mice** |
| | Table Analyzed | pgsk3a/b | | --- | --- | |  |  | | Column B | - | | vs. | vs. | | Column A | + | |  |  | | Paired t test |  | | P value | 0.0447 | | P value summary | * | | Significantly different (P < 0.05)? | Yes | | One- or two-tailed P value? | Two-tailed | | t, df | t=2.440, df=7 | | Number of pairs | 8 | |
| **Figure 5F. pGSK3α/β expression in Hippocampus of 6M PS19 mice** |
| | Table Analyzed | pGSK3a/b | | --- | --- | |  |  | | Column B | - | | vs. | vs. | | Column A | + | |  |  | | Paired t test |  | | P value | 0.1021 | | P value summary | ns | | Significantly different (P < 0.05)? | No | | One- or two-tailed P value? | One-tailed | | t, df | t=1.400, df=7 | | Number of pairs | 8 | |
| **Figure 5G. pGSK3α/β intensity in Entorhinal cortex of 6M PS19 mice** |
| | Table Analyzed | pgsk3b | | --- | --- | |  |  | | Column B | - + | | vs. | vs. | | Column A | + - | |  |  | | Unpaired t test |  | | P value | 0.0353 | | P value summary | * | | Significantly different (P < 0.05)? | Yes | | One- or two-tailed P value? | Two-tailed | | t, df | t=2.166, df=48 | |
| **Figure 5G. pGSK3α/β intensity in Hippocampal CA1 of 6M PS19 mice** |
| | Table Analyzed | pgsk3b | | --- | --- | |  |  | | Column D | - + | | vs. | vs. | | Column C | + - | |  |  | | Unpaired t test |  | | P value | 0.4238 | | P value summary | ns | | Significantly different (P < 0.05)? | No | | One- or two-tailed P value? | One-tailed | | t, df | t=0.1934, df=46 | |
| **Figure 5G. pGSK3α/β intensity in Hippocampal DG of 6M PS19 mice** |
| | Table Analyzed | pgsk3b | | --- | --- | |  |  | | Column F | - + | | vs. | vs. | | Column E | + - | |  |  | | Unpaired t test |  | | P value | 0.8034 | | P value summary | ns | | Significantly different (P < 0.05)? | No | | One- or two-tailed P value? | Two-tailed | | t, df | t=0.2504, df=46 | |  |  | |
| **Figure 5H. pCDK5 expression in Entorhinal cortex of 6M PS19 mice** |
| | Table Analyzed | pCDK5 | | --- | --- | |  |  | | Column B | - | | vs. | vs. | | Column A | + | |  |  | | Paired t test |  | | P value | 0.0201 | | P value summary | * | | Significantly different (P < 0.05)? | Yes | | One- or two-tailed P value? | Two-tailed | | t, df | t=3.140, df=6 | | Number of pairs | 7 | |
| **Figure 5I. pCDK5 expression in Hippocampus of 6M PS19 mice** |
| | Table Analyzed | pCDK5 | | --- | --- | |  |  | | Column B | - | | vs. | vs. | | Column A | + | |  |  | | Paired t test |  | | P value | 0.4949 | | P value summary | ns | | Significantly different (P < 0.05)? | No | | One- or two-tailed P value? | One-tailed | | t, df | t=0.01332, df=7 | | Number of pairs | 8 | |
| **Figure 5J. CDK5 expression in Entorhinal cortex of 6M** PS19 mice |
| | Table Analyzed | CDK5 | | --- | --- | |  |  | | Column B | - | | vs. | vs. | | Column A | + | |  |  | | Unpaired t test |  | | P value | 0.8582 | | P value summary | ns | | Significantly different (P < 0.05)? | No | | One- or two-tailed P value? | Two-tailed | | t, df | t=0.1834, df=10 | |
| **Figure 5K. CDK5 expression in Hippocampus cortex of 6M** PS19 mice |
| | Table Analyzed | CDK5 | | --- | --- | |  |  | | Column B | - | | vs. | vs. | | Column A | + | |  |  | | Unpaired t test |  | | P value | 0.4840 | | P value summary | ns | | Significantly different (P < 0.05)? | No | | One- or two-tailed P value? | One-tailed | | t, df | t=0.04122, df=10 | |
| **Figure 5L. pCDK5 intensity in Entorhinal cortex of 6M PS19 mice** |
| | Table Analyzed | pCDK5 | | --- | --- | |  |  | | Column B | - + | | vs. | vs. | | Column A | + - | |  |  | | Unpaired t test |  | | P value | 0.0022 | | P value summary | ** | | Significantly different (P < 0.05)? | Yes | | One- or two-tailed P value? | Two-tailed | | t, df | t=3.258, df=43 | |  |  | |
| **Figure 5L. pCDK5 intensity in Hippocampal CA1 of 6M PS19 mice** |
| | Table Analyzed | pCDK5 | | --- | --- | |  |  | | Column D | - + | | vs. | vs. | | Column C | + - | |  |  | | Unpaired t test |  | | P value | 0.2364 | | P value summary | ns | | Significantly different (P < 0.05)? | No | | One- or two-tailed P value? | Two-tailed | | t, df | t=1.199, df=47 | |
| **Figure 5L. pCDK5 intensity in Hippocampal DG of 6M PS19 mice** |
| | Table Analyzed | pCDK5 | | --- | --- | |  |  | | Column F | - + | | vs. | vs. | | Column E | + - | |  |  | | Unpaired t test |  | | P value | 0.0234 | | P value summary | * | | Significantly different (P < 0.05)? | Yes | | One- or two-tailed P value? | Two-tailed | | t, df | t=2.341, df=48 | |
| **Figure 6A. Iba-1 intensity in Entorhinal cortex of 6M PS19 mice** |
| | Table Analyzed | iba-1 | | --- | --- | |  |  | | Column B | - + | | vs. | vs. | | Column A | + - | |  |  | | Unpaired t test |  | | P value | 0.9853 | | P value summary | ns | | Significantly different (P < 0.05)? | No | | One- or two-tailed P value? | Two-tailed | | t, df | t=0.01857, df=45 | |
| **Figure 6A. Iba-1 intensity in Hippocampal CA1 of 6M PS19 mice** |
| | Table Analyzed | iba-1 | | --- | --- | |  |  | | Column D | - + | | vs. | vs. | | Column C | + - | |  |  | | Unpaired t test |  | | P value | 0.3174 | | P value summary | ns | | Significantly different (P < 0.05)? | No | | One- or two-tailed P value? | Two-tailed | | t, df | t=1.011, df=46 | |
| **Figure 6A. Iba-1 intensity in Hippocampal DG of 6M PS19 mice** |
| | Table Analyzed | iba-1 | | --- | --- | |  |  | | Column F | - + | | vs. | vs. | | Column E | + - | |  |  | | Unpaired t test |  | | P value | 0.2366 | | P value summary | ns | | Significantly different (P < 0.05)? | No | | One- or two-tailed P value? | Two-tailed | | t, df | t=1.199, df=46 | |
| **Figure 6A. Iba-1-(+)-cell numbers in Entorhinal cortex of 6M PS19 mice** |
| | Table Analyzed | iba-1 | | --- | --- | |  |  | | Column B | - + | | vs. | vs. | | Column A | + - | |  |  | | Unpaired t test |  | | P value | 0.7910 | | P value summary | ns | | Significantly different (P < 0.05)? | No | | One- or two-tailed P value? | Two-tailed | | t, df | t=0.2666, df=45 | |
| **Figure6. Iba-1-(+)-cell numbers in Hippocampal CA1 of 6M PS19 mice** |
| | Table Analyzed | iba-1 | | --- | --- | |  |  | | Column D | - + | | vs. | vs. | | Column C | + - | |  |  | | Unpaired t test |  | | P value | 0.8853 | | P value summary | ns | | Significantly different (P < 0.05)? | No | | One- or two-tailed P value? | Two-tailed | | t, df | t=0.1451, df=46 | |
| **Figure 6A. Iba-1-(+)-cell numbers in Hippocampal DG of 6M PS19 mice** |
| | Table Analyzed | iba-1 | | --- | --- | |  |  | | Column F | - + | | vs. | vs. | | Column E | + - | |  |  | | Unpaired t test |  | | P value | 0.6957 | | P value summary | ns | | Significantly different (P < 0.05)? | No | | One- or two-tailed P value? | Two-tailed | | t, df | t=0.3936, df=46 | |
| **Figure 6A. Iba-1-(+)-area in Entorhinal cortex of 6M PS19 mice** |
| | Table Analyzed | iba-1 | | --- | --- | |  |  | | Column B | - + | | vs. | vs. | | Column A | + - | |  |  | | Unpaired t test |  | | P value | 0.1381 | | P value summary | ns | | Significantly different (P < 0.05)? | No | | One- or two-tailed P value? | Two-tailed | | t, df | t=1.510, df=45 | |
| **Figure 6A. Iba-1-(+)-area in Hippocampal CA1 of 6M PS19 mice** |
| | Table Analyzed | iba-1 | | --- | --- | |  |  | | Column D | - + | | vs. | vs. | | Column C | + - | |  |  | | Unpaired t test |  | | P value | 0.6809 | | P value summary | ns | | Significantly different (P < 0.05)? | No | | One- or two-tailed P value? | Two-tailed | | t, df | t=0.4139, df=46 | |
| **Figure 6A. Iba-1-(+)-area in Hippocampal DG of 6M PS19 mice** |
| | Table Analyzed | iba-1 | | --- | --- | |  |  | | Column F | - + | | vs. | vs. | | Column E | + - | |  |  | | Unpaired t test |  | | P value | 0.7492 | | P value summary | ns | | Significantly different (P < 0.05)? | No | | One- or two-tailed P value? | Two-tailed | | t, df | t=0.3216, df=46 | |
| **Figure 6B. GFAP intensity in Entorhinal cortex of 6M PS19 mice** |
| | Table Analyzed | GFAP | | --- | --- | |  |  | | Column B | - + | | vs. | vs. | | Column A | + - | |  |  | | Unpaired t test |  | | P value | 0.0076 | | P value summary | ** | | Significantly different (P < 0.05)? | Yes | | One- or two-tailed P value? | Two-tailed | | t, df | t=2.798, df=45 | |
| **Figure 6B. GFAP intensity in Hippocampal CA1 of 6M PS19 mice** |
| | Table Analyzed | GFAP | | --- | --- | |  |  | | Column D | - + | | vs. | vs. | | Column C | + - | |  |  | | Unpaired t test |  | | P value | 0.1800 | | P value summary | ns | | Significantly different (P < 0.05)? | No | | One- or two-tailed P value? | Two-tailed | | t, df | t=1.361, df=46 | |
| **Figure 6B. GFAP intensity in Hippocampal DG of 6M PS19 mice** |
| | Table Analyzed | GFAP | | --- | --- | |  |  | | Column F | - + | | vs. | vs. | | Column E | + - | |  |  | | Unpaired t test |  | | P value | 0.1445 | | P value summary | ns | | Significantly different (P < 0.05)? | No | | One- or two-tailed P value? | Two-tailed | | t, df | t=1.485, df=46 | |
| **Figure 6B. GFAP-(+)-cell numbers in Entorhinal cortex of 6M PS19 mice** |
| | Table Analyzed | GFAP | | --- | --- | |  |  | | Column B | - + | | vs. | vs. | | Column A | + - | |  |  | | Unpaired t test |  | | P value | 0.0075 | | P value summary | ** | | Significantly different (P < 0.05)? | Yes | | One- or two-tailed P value? | Two-tailed | | t, df | t=2.803, df=45 | |
| **Figure 6B. GFAP-(+)-cell numbers in Hippocampal CA1 of 6M PS19 mice** |
| | Table Analyzed | GFAP | | --- | --- | |  |  | | Column D | - + | | vs. | vs. | | Column C | + - | |  |  | | Unpaired t test |  | | P value | 0.2505 | | P value summary | ns | | Significantly different (P < 0.05)? | No | | One- or two-tailed P value? | Two-tailed | | t, df | t=1.164, df=46 | |
| **Figure 6B. GFAP-(+)-cell numbers in Hippocampal DG of 6M PS19 mice** |
| | Table Analyzed | GFAP | | --- | --- | |  |  | | Column F | - + | | vs. | vs. | | Column E | + - | |  |  | | Unpaired t test |  | | P value | 0.1392 | | P value summary | ns | | Significantly different (P < 0.05)? | No | | One- or two-tailed P value? | Two-tailed | | t, df | t=1.505, df=46 | |  |  | |
| **Figure 6B. GFAP-(+)-area in Entorhinal cortex of 6M PS19 mice** |
| | Table Analyzed | GFAP | | --- | --- | |  |  | | Column B | - + | | vs. | vs. | | Column A | + - | |  |  | | Unpaired t test |  | | P value | 0.0110 | | P value summary | * | | Significantly different (P < 0.05)? | Yes | | One- or two-tailed P value? | Two-tailed | | t, df | t=2.654, df=45 | |
| **Figure 6B. GFAP-(+)-area in Hippocampal CA1 of 6M PS19 mice** |
| | Table Analyzed | GFAP | | --- | --- | |  |  | | Column D | - + | | vs. | vs. | | Column C | + - | |  |  | | Unpaired t test |  | | P value | 0.3339 | | P value summary | ns | | Significantly different (P < 0.05)? | No | | One- or two-tailed P value? | Two-tailed | | t, df | t=0.9766, df=46 | |
| **Figure 6B. GFAP-(+)-area in Hippocampal DG of 6M PS19 mice** |
| | Table Analyzed | GFAP | | --- | --- | |  |  | | Column F | - + | | vs. | vs. | | Column E | + - | |  |  | | Unpaired t test |  | | P value | 0.2467 | | P value summary | ns | | Significantly different (P < 0.05)? | No | | One- or two-tailed P value? | Two-tailed | | t, df | t=1.173, df=46 | |
| **Figure 6C. *il-1β* mRNA levels in Primary astrocyte from 6M PS19 mice** |
| | Table Analyzed | IL-1beta | | --- | --- | |  |  | | Column B | PS19+7786 | | vs. | vs. | | Column A | PS19+Veh | |  |  | | Unpaired t test |  | | P value | 0.0107 | | P value summary | * | | Significantly different (P < 0.05)? | Yes | | One- or two-tailed P value? | Two-tailed | | t, df | t=2.943, df=14 | |
| **Figure 6C. *cox-2* mRNA levels in Primary astrocyte from 6M PS19 mice** |
| | Table Analyzed | cox-2 | | --- | --- | |  |  | | Column B | PS19+7786 | | vs. | vs. | | Column A | PS19+Veh | |  |  | | Unpaired t test |  | | P value | 0.0449 | | P value summary | * | | Significantly different (P < 0.05)? | Yes | | One- or two-tailed P value? | Two-tailed | | t, df | t=2.203, df=14 | |
| **Figure 6C. *il-6* mRNA levels in Primary astrocyte from 6M PS19 mice** |
| | Table Analyzed | il-6 | | --- | --- | |  |  | | Column B | PS19+7786 | | vs. | vs. | | Column A | PS19+Veh | |  |  | | Unpaired t test |  | | P value | 0.7555 | | P value summary | ns | | Significantly different (P < 0.05)? | No | | One- or two-tailed P value? | Two-tailed | | t, df | t=0.3176, df=14 | |
| **Figure 6C. *tnf-α* mRNA levels in Primary astrocyte from 6M PS19 mice** |
| | Table Analyzed | tnf-a | | --- | --- | |  |  | | Column B | PS19+7786 | | vs. | vs. | | Column A | PS19+Veh | |  |  | | Unpaired t test |  | | P value | 0.0772 | | P value summary | ns | | Significantly different (P < 0.05)? | No | | One- or two-tailed P value? | Two-tailed | | t, df | t=1.907, df=14 | |
| **Figure 7A. p-EGFR intensity in Cortex of 3.5M 5xFAD mice** |
| | Table Analyzed | p-EGFR | | --- | --- | |  |  | | Column B | - | | vs. | vs. | | Column A | + | |  |  | | Unpaired t test |  | | P value | 0.0032 | | P value summary | ** | | Significantly different (P < 0.05)? | Yes | | One- or two-tailed P value? | Two-tailed | | t, df | t=3.201, df=30 | |
| **Figure 7A. p-EGFR intensity in Hippocampal CA1 of 3.5M 5xFAD mice** |
| | Table Analyzed | p-EGFR | | --- | --- | |  |  | | Column D | - | | vs. | vs. | | Column C | + | |  |  | | Unpaired t test |  | | P value | 0.0012 | | P value summary | ** | | Significantly different (P < 0.05)? | Yes | | One- or two-tailed P value? | Two-tailed | | t, df | t=3.568, df=30 | |
| **Figure 7A. p-EGFR intensity in Hippocampal DG of 3.5M 5xFAD mice** |
| | Table Analyzed | p-EGFR | | --- | --- | |  |  | | Column F | - | | vs. | vs. | | Column E | + | |  |  | | Unpaired t test |  | | P value | <0.0001 | | P value summary | **** | | Significantly different (P < 0.05)? | Yes | | One- or two-tailed P value? | Two-tailed | | t, df | t=4.575, df=30 | |
| **Figure 7B. Alternation % in Y maze (3M 5xFAD mice)** |
| | Table Analyzed | Y-maze | | --- | --- | |  |  | | Column A | + | | vs. | vs. | | Column B | - | |  |  | | Unpaired t test |  | | P value | 0.0390 | | P value summary | * | | Significantly different (P < 0.05)? | Yes | | One- or two-tailed P value? | Two-tailed | | t, df | t=2.278, df=14 | |
| **Figure 7B. Total arm entry numbers in Y maze (3M 5xFAD mice)** |
| | Table Analyzed | Y-maze | | --- | --- | |  |  | | Column B | - | | vs. | vs. | | Column A | + | |  |  | | Unpaired t test |  | | P value | 0.5399 | | P value summary | ns | | Significantly different (P < 0.05)? | No | | One- or two-tailed P value? | Two-tailed | | t, df | t=0.6284, df=14 | |
| **Figure 7C. Object preference in NOR training (3M 5xFAD mice**) |
| | | Table Analyzed | NORT | | --- | --- | |  |  | | Column B | Data Set-B | | vs. | vs. | | Column A | Data Set-A | |  |  | | Unpaired t test |  | | P value | 0.0582 | | P value summary | ns | | Significantly different (P < 0.05)? | No | | One- or two-tailed P value? | Two-tailed | | t, df | t=2.062, df=14 | |  | | --- | --- | --- | --- | --- | --- | --- | --- | --- | --- | --- | --- | --- | --- | --- | --- | --- | --- | --- | --- | --- | --- | --- | --- | --- | --- | |
| **Figure 7C. Object preference in NOR test (3M 5xFAD mice**) |
| | Table Analyzed | NORT | | --- | --- | |  |  | | Column E | Data Set-E | | vs. | vs. | | Column D | Data Set-D | |  |  | | Unpaired t test |  | | P value | 0.6696 | | P value summary | ns | | Significantly different (P < 0.05)? | No | | One- or two-tailed P value? | Two-tailed | | t, df | t=0.4359, df=14 | |
| **Figure 7D. Escape latency in PAT (3M 5xFAD mice**) |
| | Table Analyzed | PAT |  |  |  | | --- | --- | --- | --- | --- | |  |  |  |  |  | | Two-way RM ANOVA | Matching: Stacked |  |  |  | | Assume sphericity? | Yes |  |  |  | | Alpha | 0.05 |  |  |  | |  |  |  |  |  | | Source of Variation | % of total variation | P value | P value summary | Significant? | | Interaction | 2.366 | 0.2594 | ns | No | | Time | 38.82 | 0.0004 | *** | Yes | | Column Factor | 2.729 | 0.3579 | ns | No | | Subject | 35.83 | 0.1683 | ns | No |  | Compare each cell mean with the other cell mean in that column |  |  |  |  |  | | --- | --- | --- | --- | --- | --- | |  |  |  |  |  |  | | Number of families | 1 |  |  |  |  | | Number of comparisons per family | 2 |  |  |  |  | | Alpha | 0.05 |  |  |  |  | |  |  |  |  |  |  | | Bonferroni's multiple comparisons test | Predicted (LS) mean diff. | 95.00% CI of diff. | Significant? | Summary | Adjusted P Value | |  |  |  |  |  |  | | training day - test day |  |  |  |  |  | | Vehicle | -63.03 | -126.2 to 0.1630 | No | ns | 0.0506 | | Erlotinib | -104.4 | -167.5 to -41.17 | Yes | ** | 0.0023 |  | Compare each cell mean with the other cell mean in that row |  |  |  |  |  | | --- | --- | --- | --- | --- | --- | |  |  |  |  |  |  | | Number of families | 1 |  |  |  |  | | Number of comparisons per family | 2 |  |  |  |  | | Alpha | 0.05 |  |  |  |  | |  |  |  |  |  |  | | Bonferroni's multiple comparisons test | Mean Diff. | 95.00% CI of diff. | Significant? | Summary | Adjusted P Value | |  |  |  |  |  |  | | Vehicle - Erlotinib |  |  |  |  |  | | training day | -1.529 | -70.97 to 67.91 | No | ns | >0.9999 | | test day | -42.86 | -112.3 to 26.58 | No | ns | 0.3061 | |
| **Figure 7E. Dendritic spine numbers in AO region of Hippocampal CA1 from 3.5M 5xFAD mice** |
| | Table Analyzed | spine # | | --- | --- | |  |  | | Column B | Data Set-B | | vs. | vs. | | Column A | Data Set-A | |  |  | | Unpaired t test |  | | P value | 0.0001 | | P value summary | *** | | Significantly different (P < 0.05)? | Yes | | One- or two-tailed P value? | Two-tailed | | t, df | t=4.455, df=27 | |
| **Figure 7E. Dendritic spine numbers in BS region of Hippocampal CA1 from 3.5M 5xFAD mice** |
| | Table Analyzed | spine # | | --- | --- | |  |  | | Column E | Data Set-E | | vs. | vs. | | Column D | Data Set-D | |  |  | | Unpaired t test |  | | P value | 0.0162 | | P value summary | * | | Significantly different (P < 0.05)? | Yes | | One- or two-tailed P value? | Two-tailed | | t, df | t=2.566, df=27 | |
| **Figure 7F. Aβ plaque numbers (6E10**) in Cortex of 3.5M 5xFAD mice |
| | Table Analyzed | 6E10 | | --- | --- | |  |  | | Column A | + | | vs. | vs. | | Column B | - | |  |  | | Unpaired t test |  | | P value | 0.0005 | | P value summary | *** | | Significantly different (P < 0.05)? | Yes | | One- or two-tailed P value? | Two-tailed | | t, df | t=3.920, df=30 | |
| **Figure 7F. Aβ plaque numbers (6E10) in Hippocampal CA1 of 3.5M 5xFAD mice** |
| | Table Analyzed | 6E10 | | --- | --- | |  |  | | Column C | + | | vs. | vs. | | Column D | - | |  |  | | Unpaired t test |  | | P value | 0.0102 | | P value summary | * | | Significantly different (P < 0.05)? | Yes | | One- or two-tailed P value? | Two-tailed | | t, df | t=2.741, df=30 | |
| **Figure 7F. Aβ plaque numbers (6E10) in Hippocampal DG of 3.5M 5xFAD mice** |
| | Table Analyzed | 6E10 | | --- | --- | |  |  | | Column E | + | | vs. | vs. | | Column F | - | |  |  | | Unpaired t test |  | | P value | 0.0067 | | P value summary | ** | | Significantly different (P < 0.05)? | Yes | | One- or two-tailed P value? | Two-tailed | | t, df | t=2.911, df=30 | |
| **Figure 8A. AT8 intensity in Cortex of 3.5M** 5xFAD mice |
| | Table Analyzed | AT8 | | --- | --- | |  |  | | Column B | - | | vs. | vs. | | Column A | + | |  |  | | Unpaired t test |  | | P value | <0.0001 | | P value summary | **** | | Significantly different (P < 0.05)? | Yes | | One- or two-tailed P value? | Two-tailed | | t, df | t=4.912, df=30 | |
| **Figure 8A. AT8 intensity in Hippocampal CA1 of 3.5M 5xFAD mice** |
| | Table Analyzed | AT8 | | --- | --- | |  |  | | Column D | - | | vs. | vs. | | Column C | + | |  |  | | Unpaired t test |  | | P value | 0.0002 | | P value summary | *** | | Significantly different (P < 0.05)? | Yes | | One- or two-tailed P value? | Two-tailed | | t, df | t=4.313, df=30 | |
| **Figure 8A. AT8 intensity in Hippocampal DG of 3.5M 5xFAD mice** |
| | Table Analyzed | AT8 | | --- | --- | |  |  | | Column F | - | | vs. | vs. | | Column E | + | |  |  | | Unpaired t test |  | | P value | 0.0045 | | P value summary | ** | | Significantly different (P < 0.05)? | Yes | | One- or two-tailed P value? | Two-tailed | | t, df | t=3.076, df=30 | |
| **Figure 8B. AT100 intensity in Cortex of 3.5M 5xFAD mice** |
| | Table Analyzed | AT100 | | --- | --- | |  |  | | Column B | - | | vs. | vs. | | Column A | + | |  |  | | Unpaired t test |  | | P value | 0.3090 | | P value summary | ns | | Significantly different (P < 0.05)? | No | | One- or two-tailed P value? | Two-tailed | | t, df | t=1.035, df=30 | |
| **Figure 8B. AT100 intensity in Hippocampal CA1 of 3.5M 5xFAD mice** |
| | Table Analyzed | AT100 | | --- | --- | |  |  | | Column D | - | | vs. | vs. | | Column C | + | |  |  | | Unpaired t test |  | | P value | <0.0001 | | P value summary | **** | | Significantly different (P < 0.05)? | Yes | | One- or two-tailed P value? | Two-tailed | | t, df | t=4.747, df=30 | |
| **Figure 8B. AT100 intensity in Hippocampal DG of 3.5M 5xFAD mice** |
| | Table Analyzed | AT100 | | --- | --- | |  |  | | Column F | - | | vs. | vs. | | Column E | + | |  |  | | Unpaired t test |  | | P value | <0.0001 | | P value summary | **** | | Significantly different (P < 0.05)? | Yes | | One- or two-tailed P value? | Two-tailed | | t, df | t=5.067, df=30 | |
| **Figure 8C. DYRK1A intensity in Cortex of 3.5M 5xFAD mice** |
| | Table Analyzed | DYRK1A | | --- | --- | |  |  | | Column B | - | | vs. | vs. | | Column A | + | |  |  | | Unpaired t test |  | | P value | 0.7950 | | P value summary | ns | | Significantly different (P < 0.05)? | No | | One- or two-tailed P value? | Two-tailed | | t, df | t=0.2621, df=30 | |
| **Figure 8C. DYRK1A intensity in Hippocampal CA1 of 3.5M 5xFAD mice** |
| | Table Analyzed | DYRK1A | | --- | --- | |  |  | | Column D | - | | vs. | vs. | | Column C | + | |  |  | | Unpaired t test |  | | P value | 0.1097 | | P value summary | ns | | Significantly different (P < 0.05)? | No | | One- or two-tailed P value? | Two-tailed | | t, df | t=1.648, df=30 | |
| **Figure 8C. DYRK1A intensity in Hippocampal DG of 3.5M 5xFAD mice** |
| | Table Analyzed | DYRK1A | | --- | --- | |  |  | | Column F | - | | vs. | vs. | | Column E | + | |  |  | | Unpaired t test |  | | P value | <0.0001 | | P value summary | **** | | Significantly different (P < 0.05)? | Yes | | One- or two-tailed P value? | Two-tailed | | t, df | t=4.988, df=30 | |
| **Figure 8D. pGSK3α/β intensity in Cortex of 3.5M 5xFAD mice** |
| | Table Analyzed | pGSK3a,b | | --- | --- | |  |  | | Column B | - | | vs. | vs. | | Column A | + | |  |  | | Unpaired t test |  | | P value | 0.0074 | | P value summary | ** | | Significantly different (P < 0.05)? | Yes | | One- or two-tailed P value? | Two-tailed | | t, df | t=2.873, df=30 | |
| **Figure 8D. pGSK3α/β intensity in Hippocampal CA1 of 3.5M 5xFAD mice** |
| | Table Analyzed | pGSK3a,b | | --- | --- | |  |  | | Column D | - | | vs. | vs. | | Column C | + | |  |  | | Unpaired t test |  | | P value | 0.0160 | | P value summary | * | | Significantly different (P < 0.05)? | Yes | | One- or two-tailed P value? | Two-tailed | | t, df | t=2.552, df=30 | |
| **Figure 8D. pGSK3α/β intensity in Hippocampal DG of 3.5M 5xFAD mice** |
| | Table Analyzed | pGSK3a,b | | --- | --- | |  |  | | Column F | - | | vs. | vs. | | Column E | + | |  |  | | Unpaired t test |  | | P value | 0.0571 | | P value summary | ns | | Significantly different (P < 0.05)? | No | | One- or two-tailed P value? | Two-tailed | | t, df | t=1.979, df=30 | |
| **Figure 9C. GFAP intensity in Cortex of 3.5M WT and 5xFAD mice** |
| | Number of families | 1 |  |  |  |  |  | | --- | --- | --- | --- | --- | --- | --- | | Number of comparisons per family | 6 |  |  |  |  |  | | Alpha | 0.05 |  |  |  |  |  | |  |  |  |  |  |  |  | | Tukey's multiple comparisons test | Mean Diff. | 95.00% CI of diff. | Significant? | Summary | Adjusted P Value |  | | - vs. + | -21.73 | -67.71 to 24.24 | No | ns | 0.5984 | A-B | | - vs. - | -109.4 | -155.4 to -63.41 | Yes | **** | <0.0001 | A-C | | - vs. + | -57.51 | -103.5 to -11.54 | Yes | ** | 0.0085 | A-D | | + vs. - | -87.65 | -133.6 to -41.68 | Yes | **** | <0.0001 | B-C | | + vs. + | -35.78 | -81.75 to 10.19 | No | ns | 0.1793 | B-D | | - vs. + | 51.87 | 5.898 to 97.84 | Yes | * | 0.0210 | C-D | |
| **Figure 9C. GFAP intensity in Hippocampal CA1 of 3.5M WT and 5xFAD mice** |
| | Number of families | 1 |  |  |  |  |  | | --- | --- | --- | --- | --- | --- | --- | | Number of comparisons per family | 6 |  |  |  |  |  | | Alpha | 0.05 |  |  |  |  |  | |  |  |  |  |  |  |  | | Tukey's multiple comparisons test | Mean Diff. | 95.00% CI of diff. | Significant? | Summary | Adjusted P Value |  | | - vs. + | 4.506 | -28.73 to 37.74 | No | ns | 0.9841 | E-F | | - vs. - | -34.42 | -67.65 to -1.187 | Yes | * | 0.0396 | E-G | | - vs. + | -31.90 | -65.13 to 1.334 | No | ns | 0.0644 | E-H | | + vs. - | -38.93 | -72.16 to -5.693 | Yes | * | 0.0154 | F-G | | + vs. + | -36.40 | -69.64 to -3.172 | Yes | * | 0.0264 | F-H | | - vs. + | 2.521 | -30.71 to 35.75 | No | ns | 0.9971 | G-H | |
| **Figure 9C. GFAP intensity in Hippocampal DG of 3.5M WT and 5xFAD mice** |
| | Number of families | 1 |  |  |  |  |  | | --- | --- | --- | --- | --- | --- | --- | | Number of comparisons per family | 6 |  |  |  |  |  | | Alpha | 0.05 |  |  |  |  |  | |  |  |  |  |  |  |  | | Tukey's multiple comparisons test | Mean Diff. | 95.00% CI of diff. | Significant? | Summary | Adjusted P Value |  | | - vs. + | 6.481 | -27.84 to 40.80 | No | ns | 0.9590 | I-J | | - vs. - | -47.45 | -81.77 to -13.14 | Yes | ** | 0.0030 | I-K | | - vs. + | -59.55 | -93.87 to -25.23 | Yes | *** | 0.0001 | I-L | | + vs. - | -53.93 | -88.25 to -19.62 | Yes | *** | 0.0006 | J-K | | + vs. + | -66.03 | -100.3 to -31.71 | Yes | **** | <0.0001 | J-L | | - vs. + | -12.10 | -46.41 to 22.22 | No | ns | 0.7881 | K-L | |
| **Figure 9C. GFAP-(+)-cell numbers in Cortex of 3.5M WT and 5xFAD mice** |
| | Number of families | 1 |  |  |  |  |  | | --- | --- | --- | --- | --- | --- | --- | | Number of comparisons per family | 6 |  |  |  |  |  | | Alpha | 0.05 |  |  |  |  |  | |  |  |  |  |  |  |  | | Tukey's multiple comparisons test | Mean Diff. | 95.00% CI of diff. | Significant? | Summary | Adjusted P Value |  | | - vs. + | -136961 | -687697 to 413775 | No | ns | 0.9126 | A-B | | - vs. - | -1897963 | -2448700 to -1347227 | Yes | **** | <0.0001 | A-C | | - vs. + | -1985311 | -2536047 to -1434575 | Yes | **** | <0.0001 | A-D | | + vs. - | -1761003 | -2311739 to -1210267 | Yes | **** | <0.0001 | B-C | | + vs. + | -1848350 | -2399086 to -1297614 | Yes | **** | <0.0001 | B-D | | - vs. + | -87348 | -638084 to 463388 | No | ns | 0.9750 | C-D | |
| **Figure 9C. GFAP-(+)-cell numbers in Hippocampal CA1 of 3.5M WT and 5xFAD mice** |
| | Number of families | 1 |  |  |  |  |  | | --- | --- | --- | --- | --- | --- | --- | | Number of comparisons per family | 6 |  |  |  |  |  | | Alpha | 0.05 |  |  |  |  |  | |  |  |  |  |  |  |  | | Tukey's multiple comparisons test | Mean Diff. | 95.00% CI of diff. | Significant? | Summary | Adjusted P Value |  | | - vs. + | -695348 | -1597490 to 206793 | No | ns | 0.1862 | E-F | | - vs. - | -1937683 | -2839825 to -1035541 | Yes | **** | <0.0001 | E-G | | - vs. + | -1776553 | -2678695 to -874411 | Yes | **** | <0.0001 | E-H | | + vs. - | -1242335 | -2144477 to -340193 | Yes | ** | 0.0031 | F-G | | + vs. + | -1081205 | -1983347 to -179063 | Yes | * | 0.0126 | F-H | | - vs. + | 161130 | -741012 to 1063272 | No | ns | 0.9649 | G-H | |
| **Figure 9C. GFAP-(+)-cell numbers in Hippocampal DG of 3.5M WT and 5xFAD mice** |
| | Number of families | 1 |  |  |  |  |  | | --- | --- | --- | --- | --- | --- | --- | | Number of comparisons per family | 6 |  |  |  |  |  | | Alpha | 0.05 |  |  |  |  |  | |  |  |  |  |  |  |  | | Tukey's multiple comparisons test | Mean Diff. | 95.00% CI of diff. | Significant? | Summary | Adjusted P Value |  | | - vs. + | -466785 | -1650834 to 717264 | No | ns | 0.7257 | I-J | | - vs. - | -2087677 | -3271726 to -903628 | Yes | *** | 0.0001 | I-K | | - vs. + | -1774218 | -2958267 to -590169 | Yes | ** | 0.0011 | I-L | | + vs. - | -1620891 | -2804941 to -436842 | Yes | ** | 0.0033 | J-K | | + vs. + | -1307433 | -2491482 to -123383 | Yes | * | 0.0249 | J-L | | - vs. + | 313459 | -870590 to 1497508 | No | ns | 0.8968 | K-L | |
| **Figure 9C. GFAP-(+)-area in Cortex of 3.5M WT and 5xFAD mice** |
| | Number of families | 1 |  |  |  |  |  | | --- | --- | --- | --- | --- | --- | --- | | Number of comparisons per family | 6 |  |  |  |  |  | | Alpha | 0.05 |  |  |  |  |  | |  |  |  |  |  |  |  | | Tukey's multiple comparisons test | Mean Diff. | 95.00% CI of diff. | Significant? | Summary | Adjusted P Value |  | | - vs. + | -0.1974 | -1.008 to 0.6131 | No | ns | 0.9173 | A-B | | - vs. - | -2.196 | -3.006 to -1.385 | Yes | **** | <0.0001 | A-C | | - vs. + | -1.399 | -2.209 to -0.5881 | Yes | *** | 0.0001 | A-D | | + vs. - | -1.999 | -2.809 to -1.188 | Yes | **** | <0.0001 | B-C | | + vs. + | -1.201 | -2.012 to -0.3907 | Yes | ** | 0.0013 | B-D | | - vs. + | 0.7973 | -0.01319 to 1.608 | No | ns | 0.0555 | C-D | |
| **Figure 9C. GFAP-(+)-area in Hippocampal CA1 of 3.5M WT and 5xFAD mice** |
| | Number of families | 1 |  |  |  |  |  | | --- | --- | --- | --- | --- | --- | --- | | Number of comparisons per family | 6 |  |  |  |  |  | | Alpha | 0.05 |  |  |  |  |  | |  |  |  |  |  |  |  | | Tukey's multiple comparisons test | Mean Diff. | 95.00% CI of diff. | Significant? | Summary | Adjusted P Value |  | | - vs. + | -0.03113 | -2.771 to 2.709 | No | ns | >0.9999 | E-F | | - vs. - | -2.786 | -5.526 to -0.04637 | Yes | * | 0.0448 | E-G | | - vs. + | -2.718 | -5.457 to 0.02207 | No | ns | 0.0526 | E-H | | + vs. - | -2.755 | -5.495 to -0.01525 | Yes | * | 0.0482 | F-G | | + vs. + | -2.687 | -5.426 to 0.05319 | No | ns | 0.0566 | F-H | | - vs. + | 0.06844 | -2.671 to 2.808 | No | ns | 0.9999 | G-H | |
| **Figure 9C. GFAP-(+)-area in Hippocampal DG of 3.5M WT and 5xFAD mice** |
| | Number of families | 1 |  |  |  |  |  | | --- | --- | --- | --- | --- | --- | --- | | Number of comparisons per family | 6 |  |  |  |  |  | | Alpha | 0.05 |  |  |  |  |  | |  |  |  |  |  |  |  | | Tukey's multiple comparisons test | Mean Diff. | 95.00% CI of diff. | Significant? | Summary | Adjusted P Value |  | | - vs. + | 0.07437 | -2.712 to 2.861 | No | ns | 0.9999 | I-J | | - vs. - | -4.243 | -7.029 to -1.457 | Yes | *** | 0.0009 | I-K | | - vs. + | -5.723 | -8.509 to -2.937 | Yes | **** | <0.0001 | I-L | | + vs. - | -4.318 | -7.104 to -1.531 | Yes | *** | 0.0007 | J-K | | + vs. + | -5.797 | -8.584 to -3.011 | Yes | **** | <0.0001 | J-L | | - vs. + | -1.480 | -4.266 to 1.306 | No | ns | 0.5022 | K-L | |
| **Figure 9F. CXCL10 intensity in Cortex of 3.5M WT and 5xFAD mice** |
| | Table Analyzed | CXCL10-intensity-cor | | --- | --- | | Data sets analyzed | A-D | |  |  | | ANOVA summary |  | | F | 9.384 | | P value | <0.0001 | | P value summary | **** | | Significant diff. among means (P < 0.05)? | Yes | | R square | 0.3557 |  | Number of families | 1 |  |  |  |  |  | | --- | --- | --- | --- | --- | --- | --- | | Number of comparisons per family | 6 |  |  |  |  |  | | Alpha | 0.05 |  |  |  |  |  | |  |  |  |  |  |  |  | | Tukey's multiple comparisons test | Mean Diff. | 95.00% CI of diff. | Significant? | Summary | Adjusted P Value |  | | - vs. + | -6.209 | -50.30 to 37.88 | No | ns | 0.9820 | A-B | | - vs. - | -72.55 | -113.8 to -31.30 | Yes | *** | 0.0001 | A-C | | - vs. + | -32.42 | -74.25 to 9.405 | No | ns | 0.1806 | A-D | | + vs. - | -66.34 | -107.6 to -25.10 | Yes | *** | 0.0005 | B-C | | + vs. + | -26.21 | -68.04 to 15.61 | No | ns | 0.3528 | B-D | | - vs. + | 40.12 | 1.310 to 78.94 | Yes | * | 0.0402 | C-D | |
| **Figure 9F. CXCL10 intensity in Hippocampal CA1 of 3.5M WT and 5xFAD mice** |
| | Number of families | 1 |  |  |  |  |  | | --- | --- | --- | --- | --- | --- | --- | | Number of comparisons per family | 6 |  |  |  |  |  | | Alpha | 0.05 |  |  |  |  |  | |  |  |  |  |  |  |  | | Tukey's multiple comparisons test | Mean Diff. | 95.00% CI of diff. | Significant? | Summary | Adjusted P Value |  | | - vs. + | 4.675 | -25.90 to 35.25 | No | ns | 0.9772 | A-B | | - vs. - | -52.21 | -80.81 to -23.60 | Yes | **** | <0.0001 | A-C | | - vs. + | -23.00 | -51.61 to 5.600 | No | ns | 0.1558 | A-D | | + vs. - | -56.88 | -85.48 to -28.28 | Yes | **** | <0.0001 | B-C | | + vs. + | -27.68 | -56.28 to 0.9248 | No | ns | 0.0612 | B-D | | - vs. + | 29.20 | 2.723 to 55.68 | Yes | * | 0.0253 | C-D | |
| **Figure 9F. CXCL10 intensity in Hippocampal DG of 3.5M WT and 5xFAD mice** |
| | Number of families | 1 | |  | |  |  |  |  | | --- | --- | --- | --- | --- | --- | --- | --- | --- | | Number of comparisons per family | 6 | |  | |  |  |  |  | | Alpha | 0.05 | |  | |  |  |  |  | |  |  | |  | |  |  |  |  | | Tukey's multiple comparisons test | Mean Diff. | | 95.00% CI of diff. | | Significant? | Summary | Adjusted P Value |  | | - vs. + | -0.8495 | | -39.00 to 37.31 | | No | ns | >0.9999 | A-B | | - vs. - | -54.45 | | -90.14 to -18.76 | | Yes | *** | 0.0010 | A-C | | - vs. + | -28.23 | | -63.92 to 7.457 | | No | ns | 0.1668 | A-D | | + vs. - | -53.60 | | -89.29 to -17.91 | | Yes | ** | 0.0012 | B-C | | + vs. + | -27.38 | | -63.08 to 8.307 | | No | ns | 0.1881 | B-D | | - vs. + | 26.22 | | -6.825 to 59.26 | | No | ns | 0.1647 | C-D | | Table Analyzed | | CXCL10-intensity-DG | |  | | | | | |  | |  | |  | | | | | | Column D | | + | |  | | | | | | vs. | | vs. | |  | | | | | | Column C | | - | |  | | | | | |  | |  | |  | | | | | | Unpaired t test | |  | |  | | | | | | P value | | 0.0528 | |  | | | | | | P value summary | | ns | |  | | | | | | Significantly different (P < 0.05)? | | No | |  | | | | | | One- or two-tailed P value? | | Two-tailed | |  | | | | | | t, df | | t=2.017, df=30 | |  | | | | | |
| **Figure 9G. *cxcl10* mRNA levels in Primary astrocyte from 5xFAD mice** |
| | Table Analyzed | PAC | | --- | --- | |  |  | | Column B | - + | | vs. | vs. | | Column A | + - | |  |  | | Unpaired t test |  | | P value | 0.0328 | | P value summary | * | | Significantly different (P < 0.05)? | Yes | | One- or two-tailed P value? | Two-tailed | | t, df | t=2.369, df=14 | |
| **Figure 9G. *gbp2* mRNA levels in Primary astrocyte from 5xFAD mice** |
| | Table Analyzed | PAC | | --- | --- | |  |  | | Column D | - + | | vs. | vs. | | Column C | + - | |  |  | | Unpaired t test |  | | P value | 0.0060 | | P value summary | ** | | Significantly different (P < 0.05)? | Yes | | One- or two-tailed P value? | Two-tailed | | t, df | t=3.231, df=14 | |
| **Figure 9G. *s100a10* mRNA levels in Primary astrocyte from 5xFAD mice** |
| | Table Analyzed | PAC | | --- | --- | |  |  | | Column F | - + | | vs. | vs. | | Column E | + - | |  |  | | Unpaired t test |  | | P value | 0.4982 | | P value summary | ns | | Significantly different (P < 0.05)? | No | | One- or two-tailed P value? | Two-tailed | | t, df | t=0.6955, df=14 | |
| **Figure 9H. *il-1β* mRNA levels in Primary astrocyte from 5xFAD mice** |
| | Table Analyzed | PAC | | --- | --- | |  |  | | Column B | - + | | vs. | vs. | | Column A | + - | |  |  | | Unpaired t test |  | | P value | 0.0319 | | P value summary | * | | Significantly different (P < 0.05)? | Yes | | One- or two-tailed P value? | Two-tailed | | t, df | t=2.383, df=14 | |
| **Figure 9H. *cox-2* mRNA levels in Primary astrocyte from 5xFAD mice** |
| | Table Analyzed | PAC | | --- | --- | |  |  | | Column D | - + | | vs. | vs. | | Column C | + - | |  |  | | Unpaired t test |  | | P value | 0.0005 | | P value summary | *** | | Significantly different (P < 0.05)? | Yes | | One- or two-tailed P value? | Two-tailed | | t, df | t=4.480, df=14 | |
| **Figure 9H. *il-6* mRNA levels in Primary astrocyte from 5xFAD mice** |
| | Table Analyzed | PAC | | --- | --- | |  |  | | Column B | - + | | vs. | vs. | | Column A | + - | |  |  | | Unpaired t test |  | | P value | 0.0378 | | P value summary | * | | Significantly different (P < 0.05)? | Yes | | One- or two-tailed P value? | Two-tailed | | t, df | t=2.294, df=14 | |
| **Figure 9H. *tnf-α* mRNA levels in Primary astrocyte from 5xFAD mice** |
| | Table Analyzed | PAC | | --- | --- | |  |  | |  |  | | Column D | - + | | vs. | vs. | | Column C | + - | |  |  | | Unpaired t test |  | | P value | 0.0388 | | P value summary | * | | Significantly different (P < 0.05)? | Yes | | One- or two-tailed P value? | Two-tailed | | t, df | t=2.280, df=14 | |  |  | |
| **Figure 9I. IL-1β protein levels in Primary astrocyte from 5xFAD mice** |
| | Table Analyzed | PAC | | --- | --- | |  |  | | Column B | - + | | vs. | vs. | | Column A | + - | |  |  | | Unpaired t test |  | | P value | 0.0065 | | P value summary | ** | | Significantly different (P < 0.05)? | Yes | | One- or two-tailed P value? | Two-tailed | | t, df | t=3.420, df=10 | |
| **Figure 9I. COX-2 protein levels in Primary astrocyte from 5xFAD mice** |
| | Table Analyzed | PAC | | --- | --- | |  |  | | Column B | - + | | vs. | vs. | | Column A | + - | |  |  | | Unpaired t test |  | | P value | 0.0228 | | P value summary | * | | Significantly different (P < 0.05)? | Yes | | One- or two-tailed P value? | Two-tailed | | t, df | t=2.489, df=18 | |
| **Figure 9I. IL-6 protein levels in Primary astrocyte from 5xFAD mice** |
| | Table Analyzed | PAC | | --- | --- | |  |  | | Column B | - + | | vs. | vs. | | Column A | + - | |  |  | | Unpaired t test |  | | P value | 0.0399 | | P value summary | * | | Significantly different (P < 0.05)? | Yes | | One- or two-tailed P value? | Two-tailed | | t, df | t=2.360, df=10 | |
| **Figure 9Ia. TNF-α protein levels in Primary astrocyte from 5xFAD mice** |
| | Table Analyzed | PAC | | --- | --- | |  |  | | Column B | - + | | vs. | vs. | | Column A | + - | |  |  | | Unpaired t test |  | | P value | 0.0312 | | P value summary | * | | Significantly different (P < 0.05)? | Yes | | One- or two-tailed P value? | One-tailed | | t, df | t=1.987, df=18 | |
| **Supple Figure 1B. Aβ plaque numbers (4G8) in cortex from 3.5M** 5xFAD mice |
| | Table Analyzed | 4G8 | | --- | --- | |  |  | | Column B | - | | vs. | vs. | | Column A | + | |  |  | | Unpaired t test |  | | P value | <0.0001 | | P value summary | **** | | Significantly different (P < 0.05)? | Yes | | One- or two-tailed P value? | Two-tailed | | t, df | t=5.193, df=30 | |
| **Supple Figure 1B. Aβ plaque numbers (4G8) in Hippocampal CA1 from 3.5M 5xFAD mice** |
| | Table Analyzed | 4G8 | | --- | --- | |  |  | | Column D | - | | vs. | vs. | | Column C | + | |  |  | | Unpaired t test |  | | P value | 0.0030 | | P value summary | ** | | Significantly different (P < 0.05)? | Yes | | One- or two-tailed P value? | Two-tailed | | t, df | t=3.232, df=30 | |
| **Supple Figure 1B. Aβ plaque numbers (4G8) in Hippocampal DG from 3.5M 5xFAD mice** |
| | Table Analyzed | 4G8 | | --- | --- | |  |  | | Column F | - | | vs. | vs. | | Column E | + | |  |  | | Unpaired t test |  | | P value | 0.0009 | | P value summary | *** | | Significantly different (P < 0.05)? | Yes | | One- or two-tailed P value? | Two-tailed | | t, df | t=3.678, df=30 | |
| **Supple Figure 2C. Iba-1 intensity in Cortex from 3.5M WT and 5xFAD mice** |
| | Number of families | 1 |  |  |  |  |  | | --- | --- | --- | --- | --- | --- | --- | | Number of comparisons per family | 6 |  |  |  |  |  | | Alpha | 0.05 |  |  |  |  |  | |  |  |  |  |  |  |  | | Tukey's multiple comparisons test | Mean Diff. | 95.00% CI of diff. | Significant? | Summary | Adjusted P Value |  | | - vs. + | -9.745 | -35.13 to 15.64 | No | ns | 0.7417 | A-B | | - vs. - | -76.45 | -101.8 to -51.06 | Yes | **** | <0.0001 | A-C | | - vs. + | -51.48 | -76.87 to -26.10 | Yes | **** | <0.0001 | A-D | | + vs. - | -66.70 | -92.09 to -41.31 | Yes | **** | <0.0001 | B-C | | + vs. + | -41.74 | -67.13 to -16.35 | Yes | *** | 0.0003 | B-D | | - vs. + | 24.96 | -0.4247 to 50.35 | No | ns | 0.0556 | C-D | |
| **Supple Figure 2C. Iba-1 intensity in Hippocampal CA1 from 3.5M WT and 5xFAD mice** |
| | Number of families | 1 |  |  |  |  |  | | --- | --- | --- | --- | --- | --- | --- | | Number of comparisons per family | 6 |  |  |  |  |  | | Alpha | 0.05 |  |  |  |  |  | |  |  |  |  |  |  |  | | Tukey's multiple comparisons test | Mean Diff. | 95.00% CI of diff. | Significant? | Summary | Adjusted P Value |  | | - vs. + | 5.946 | -28.27 to 40.16 | No | ns | 0.9675 | E-F | | - vs. - | -77.48 | -112.3 to -42.70 | Yes | **** | <0.0001 | E-G | | - vs. + | -67.02 | -101.2 to -32.81 | Yes | **** | <0.0001 | E-H | | + vs. - | -83.43 | -118.2 to -48.65 | Yes | **** | <0.0001 | F-G | | + vs. + | -72.97 | -107.2 to -38.75 | Yes | **** | <0.0001 | F-H | | - vs. + | 10.46 | -24.32 to 45.24 | No | ns | 0.8564 | G-H | |
| **Supple Figure 2C. Iba-1 intensity in Hippocampal DG from 3.5M WT and 5xFAD mice** |
| | Number of families | 1 |  |  |  |  |  | | --- | --- | --- | --- | --- | --- | --- | | Number of comparisons per family | 6 |  |  |  |  |  | | Alpha | 0.05 |  |  |  |  |  | |  |  |  |  |  |  |  | | Tukey's multiple comparisons test | Mean Diff. | 95.00% CI of diff. | Significant? | Summary | Adjusted P Value |  | | - vs. + | -3.098 | -36.13 to 29.94 | No | ns | 0.9946 | I-J | | - vs. - | -97.78 | -131.4 to -64.20 | Yes | **** | <0.0001 | I-K | | - vs. + | -64.94 | -97.97 to -31.90 | Yes | **** | <0.0001 | I-L | | + vs. - | -94.68 | -128.3 to -61.10 | Yes | **** | <0.0001 | J-K | | + vs. + | -61.84 | -94.87 to -28.81 | Yes | **** | <0.0001 | J-L | | - vs. + | 32.84 | -0.7382 to 66.42 | No | ns | 0.0575 | K-L | |
| **Supple Figure 2C. Iba-1-(+)-cell numbers in Cortex from 3.5M WT and** 5xFAD mice |
| | Number of families | 1 |  |  |  |  |  | | --- | --- | --- | --- | --- | --- | --- | | Number of comparisons per family | 6 |  |  |  |  |  | | Alpha | 0.05 |  |  |  |  |  | |  |  |  |  |  |  |  | | Tukey's multiple comparisons test | Mean Diff. | 95.00% CI of diff. | Significant? | Summary | Adjusted P Value |  | | - vs. + | -3.311 | -99.14 to 92.52 | No | ns | 0.9997 | A-B | | - vs. - | -242.3 | -338.1 to -146.5 | Yes | **** | <0.0001 | A-C | | - vs. + | -225.4 | -321.2 to -129.5 | Yes | **** | <0.0001 | A-D | | + vs. - | -239.0 | -334.8 to -143.2 | Yes | **** | <0.0001 | B-C | | + vs. + | -222.0 | -317.9 to -126.2 | Yes | **** | <0.0001 | B-D | | - vs. + | 16.94 | -78.89 to 112.8 | No | ns | 0.9659 | C-D | |
| **Supple Figure 2C. Iba-1-(+)-cell numbers in Hippocampal CA1 from 3.5M WT and 5xFAD mice** |
| | Number of families | 1 |  |  |  |  |  | | --- | --- | --- | --- | --- | --- | --- | | Number of comparisons per family | 6 |  |  |  |  |  | | Alpha | 0.05 |  |  |  |  |  | |  |  |  |  |  |  |  | | Tukey's multiple comparisons test | Mean Diff. | 95.00% CI of diff. | Significant? | Summary | Adjusted P Value |  | | - vs. + | 63.41 | -15.57 to 142.4 | No | ns | 0.1579 | E-F | | - vs. - | -204.7 | -285.0 to -124.4 | Yes | **** | <0.0001 | E-G | | - vs. + | -130.8 | -209.8 to -51.84 | Yes | *** | 0.0003 | E-H | | + vs. - | -268.1 | -348.4 to -187.9 | Yes | **** | <0.0001 | F-G | | + vs. + | -194.2 | -273.2 to -115.2 | Yes | **** | <0.0001 | F-H | | - vs. + | 73.91 | -6.368 to 154.2 | No | ns | 0.0817 | G-H | |
| **Supple Figure 2C. Iba-1-(+)-cell numbers in Hippocampal DG from 3.5M WT and 5xFAD mice** |
| | Number of families | 1 |  |  |  |  |  | | --- | --- | --- | --- | --- | --- | --- | | Number of comparisons per family | 6 |  |  |  |  |  | | Alpha | 0.05 |  |  |  |  |  | |  |  |  |  |  |  |  | | Tukey's multiple comparisons test | Mean Diff. | 95.00% CI of diff. | Significant? | Summary | Adjusted P Value |  | | - vs. + | 148.5 | -79.02 to 376.1 | No | ns | 0.3197 | I-J | | - vs. - | -352.5 | -583.9 to -121.2 | Yes | *** | 0.0009 | I-K | | - vs. + | -577.0 | -804.6 to -349.4 | Yes | **** | <0.0001 | I-L | | + vs. - | -501.1 | -732.4 to -269.8 | Yes | **** | <0.0001 | J-K | | + vs. + | -725.6 | -953.1 to -498.0 | Yes | **** | <0.0001 | J-L | | - vs. + | -224.5 | -455.8 to 6.866 | No | ns | 0.0603 | K-L | |
| **Supple Figure 2C. Iba-1-(+)-area in Cortex from 3.5M WT and 5xFAD mice** |
| | Number of families | 1 |  |  |  |  |  | | --- | --- | --- | --- | --- | --- | --- | | Number of comparisons per family | 6 |  |  |  |  |  | | Alpha | 0.05 |  |  |  |  |  | |  |  |  |  |  |  |  | | Tukey's multiple comparisons test | Mean Diff. | 95.00% CI of diff. | Significant? | Summary | Adjusted P Value |  | | - vs. + | 0.04269 | -0.4665 to 0.5518 | No | ns | 0.9961 | A-B | | - vs. - | -1.370 | -1.879 to -0.8604 | Yes | **** | <0.0001 | A-C | | - vs. + | -1.062 | -1.571 to -0.5525 | Yes | **** | <0.0001 | A-D | | + vs. - | -1.412 | -1.921 to -0.9031 | Yes | **** | <0.0001 | B-C | | + vs. + | -1.104 | -1.613 to -0.5952 | Yes | **** | <0.0001 | B-D | | - vs. + | 0.3079 | -0.2012 to 0.8171 | No | ns | 0.3874 | C-D | |
| **Supple Figure 2C. Iba-1-(+)-area in Hippocampal CA1 from 3.5M WT and 5xFAD mice** |
| | Number of families | 1 |  |  |  |  |  | | --- | --- | --- | --- | --- | --- | --- | | Number of comparisons per family | 6 |  |  |  |  |  | | Alpha | 0.05 |  |  |  |  |  | |  |  |  |  |  |  |  | | Tukey's multiple comparisons test | Mean Diff. | 95.00% CI of diff. | Significant? | Summary | Adjusted P Value |  | | - vs. + | 0.3658 | -0.1455 to 0.8771 | No | ns | 0.2428 | E-F | | - vs. - | -1.358 | -1.878 to -0.8384 | Yes | **** | <0.0001 | E-G | | - vs. + | -1.077 | -1.589 to -0.5660 | Yes | **** | <0.0001 | E-H | | + vs. - | -1.724 | -2.244 to -1.204 | Yes | **** | <0.0001 | F-G | | + vs. + | -1.443 | -1.954 to -0.9318 | Yes | **** | <0.0001 | F-H | | - vs. + | 0.2808 | -0.2389 to 0.8006 | No | ns | 0.4869 | G-H | |
| **Supple Figure 2C. Iba-1-(+)-area in Hippocampal DG from 3.5M WT and 5xFAD mice** |
| | Number of families | 1 |  |  |  |  |  | | --- | --- | --- | --- | --- | --- | --- | | Number of comparisons per family | 6 |  |  |  |  |  | | Alpha | 0.05 |  |  |  |  |  | |  |  |  |  |  |  |  | | Tukey's multiple comparisons test | Mean Diff. | 95.00% CI of diff. | Significant? | Summary | Adjusted P Value |  | | - vs. + | 0.4091 | -0.6948 to 1.513 | No | ns | 0.7614 | I-J | | - vs. - | -3.160 | -4.283 to -2.038 | Yes | **** | <0.0001 | I-K | | - vs. + | -2.266 | -3.370 to -1.162 | Yes | **** | <0.0001 | I-L | | + vs. - | -3.570 | -4.692 to -2.447 | Yes | **** | <0.0001 | J-K | |  |  |  |  |  |  |  | | + vs. + | -2.675 | -3.779 to -1.571 | Yes | **** | <0.0001 | J-L | | - vs. + | 0.8942 | -0.2279 to 2.016 | No | ns | 0.1629 | K-L | |
